# Validation of quantitative real-time PCR reference genes and spatial expression profiles of detoxication-related genes under pesticide induction in honey bee, *Apis mellifera*

**DOI:** 10.1371/journal.pone.0277455

**Published:** 2022-11-10

**Authors:** YeongHo Kim, Hyemin Kim, JooHeon Cha, Si Hyeock Lee, Young Ho Kim

**Affiliations:** 1 Department of Ecological Science, Kyungpook National University, Sangju, Gyeongbuk, Republic of Korea; 2 Department of Applied Biology, Kyungpook National University, Sangju, Gyeongbuk, Republic of Korea; 3 Department of Agricultural Biotechnology, Seoul National University, Seoul, Republic of Korea; USDA Agricultural Research Service, UNITED STATES

## Abstract

Recently, pesticides have been suggested to be one of the factors responsible for the large-scale decline in honey bee populations, including colony collapse disorder. The identification of the genes that respond to pesticide exposure based on their expression is essential for understanding the xenobiotic detoxification metabolism in honey bees. For the accurate determination of target gene expression by quantitative real-time PCR, the expression stability of reference genes should be validated in honey bees exposed to various pesticides. Therefore, in this study, to select the optimal reference genes, we analyzed the amplification efficiencies of five candidate reference genes (*RPS5*, *RPS18*, *GAPDH*, *ARF1*, and *RAD1a*) and their expression stability values using four programs (geNorm, NormFinder, BestKeeper, and RefFinder) across samples of five body parts (head, thorax, gut, fat body, and carcass) from honey bees exposed to seven pesticides (acetamiprid, imidacloprid, flupyradifurone, fenitrothion, carbaryl, amitraz, and bifenthrin). Among these five candidate genes, a combination of *RAD1a* and *RPS18* was suggested for target gene normalization. Subsequently, expression levels of six genes (*AChE1*, *CYP9Q1*, *CYP9Q2*, *CYP9Q3*, *CAT*, and *SOD1*) were normalized with a combination of *RAD1a* and *RPS18* in the different body parts from honey bees exposed to pesticides. Among the six genes in the five body parts, the expression of *SOD1* in the head, fat body, and carcass was significantly induced by six pesticides. In addition, among seven pesticides, flupyradifurone statistically induced expression levels of five genes in the fat body.

## Introduction

Pesticides are indispensable in the agricultural industry for pest control, thereby reducing the loss of agricultural products and improving the yield and quality of food [1–3]. Without the use of pesticides, 78%, 54%, and 32% losses have been estimated in fruit, vegetable, and cereal production, respectively [4]. In 2018 and 2019, nearly 4.2 million metric tons of pesticides were used in the world, and the U.S. used approximately 0.4 million metric tons of pesticides [5].

Despite the benefits, pesticides can be toxic to other organisms, including birds, fish, and non-target insects [4]. Honey bees are particularly affected by unintentional exposure to pesticides during foraging. The phenomenon of rapidly declining honey bee colonies, resulting in 30–90% beehive disappearance, is known as colony collapse disorder (CCD) since its first report in the United States in late 2006 [6]. Although various factors, such as viruses, fungi, parasitic mites, limited floral resources, climate change, and the combination of these stressors have been suggested as reasons for CCD [7–11], neonicotinoid pesticides, including imidacloprid and clothianidin, have been widely reported to be the main factor causing CCD [6, 12]. CCD has been predominantly reported in North America and Europe, but honey bee colonies are also damaged by pesticide exposure due to agricultural activities on numerous other continents. In addition, the application of acaricides to control *Varroa* mites causes severe damage to honey bee colonies [13]. These pesticides can have destructive effects on honey bees, affecting immune system function, learning ability, memory, foraging behavior, and odor discrimination [14, 15], which in severe cases can have detrimental consequences for the colony [16, 17].

Although foragers are more frequently exposed to pesticides, the whole colony, including nurse bees, is also under the threat of agricultural chemicals as pesticide-contaminated nectar and pollen are delivered to the entire colony through foraging activities [18–22]. Considering that pesticides have been suggested to be a factor in large-scale honey bee decline including CCD, and nurse bees are also possibly exposed to pesticides, the xenobiotic detoxification metabolism in nurse bees should be comprehensively understood by the precise determination of target gene expression levels. In addition, the genes that are significantly increased or reduced in expression because of pesticide exposure can serve as biological markers for the analysis of damage to honey bees by pesticides. Quantitative real-time PCR (qRT-PCR) is the most extensively used method for gene expression analysis because of its rapid speed, high sensitivity, reproducibility, and accuracy. To ensure accurate normalization of target gene expression, the selection of optimal reference genes that are stably expressed under various conditions should be prioritized [23–25]. In previous studies, the suitability of the reference gene was evaluated under various conditions in honey bees, including developmental stages [26, 27], labor/seasons [28], bacterial challenge [29], tissues/seasons [30], and pesticide treatment [31, 32].

In the present study, to select suitable reference genes, we chose five candidate reference genes including 40S ribosomal protein S5 (*RPS5*), 40S ribosomal protein S18 (*RPS18*), glyceraldehyde-3-phosphate dehydrogenase (*GAPDH*), ADP-ribosylation factor 1 (*ARF1*), and Ras-related protein Rab-1A (*RAD1a*), which have been previously used as reference genes in honey bee studies [28, 30, 32]. Previous studies showed that honey bee is accidently exposed to various pesticides, and exposure of these pesticides negatively affect honey bee colonies, therefore, we chose seven pesticides (neonicotinoids: acetamiprid and imidacloprid, butanolide: flupyradifurone, organophosphate: fenitrothion, carbamate: carbaryl, formamidine: amitraz, and pyrethroid: bifenthrin) [33–38]. In addition, considering that different tissues might involve different detoxification mechanisms [39], we dissected five body parts (head, thorax, gut, fat body, and carcass) from honey bees treated with the seven pesticides. Then, expression stabilities of five reference genes were determined using their C_q_ distribution and four programs (geNorm, NormFinder, BestKeeper, and RefFinder). In addition, we compared expression levels of acetylcholinesterase 1 (*AChE1*) normalized with different combinations of reference genes and single genes to select common reference gene(s) for target gene normalization across different body parts of honey bees exposed to various pesticides.

After the selection of suitable reference gene(s), transcription levels of genes, including *AChE1*, cytochrome P450 monooxygenases (*CYPs*), superoxide dismutase (*SOD*), and catalase (*CAT*), putatively involved in the chemical detoxification system were investigated by qRT-PCR using selected reference genes in the five body parts from honey bees exposed to the seven pesticides. The honey bee *AChE1* has been suggested to be involved in various stress responses [40, 41]. In particular, *AChE1* provides chemical defense against organophosphate (OP) and carbamate (CB) insecticides in honey bees [42]. *CYPs* are well-known enzymes involved in the metabolic detoxification of pyrethroids [43], and *CYP9Q* genes contribute to the metabolic detoxification of tau-fluvalinate, coumaphos, and neonicotinoids in honey bees [44, 45]. Considering that a lot of energy is required for pesticide detoxification, which leads to increased reactive oxygen species (ROS) production [46], the upregulation of antioxidant genes, such as *SOD* and *CAT*, is an essential physiological response to prevent ROS-mediated damage [47].

Therefore, to understand the physiological response in honey bees under various pesticide exposures, we selected optimal reference genes and determined the expression levels of *AChE1*, *CYP9Q1*, *CYP9Q2*, *CYP9Q3*, *SOD1*, and *CAT*. In addition, expression profiles of these genes increased or reduced after exposure to different pesticides in different body parts of honey bees will be expected to be used as possible molecular markers to identify the pesticides damaging honey bee colonies.

## Materials and methods

### Honey bee sample preparation and pesticide exposure

Italian hybrid honey bee (*Apis mellifera*) colonies were maintained in an experimental apiary (36° 36′ 69. 09″ N, 128° 11′ 70.42″ E) in Sangju-si, Gyeongsangbuk-do, Republic of Korea, with no miticide treatment. Adult nurse bees aged five to ten days old were used in the experiment [48].

To evaluate the toxicity of the pesticides, the analytical standard grade of seven pesticides (neonicotinoids: acetamiprid and imidacloprid, butanolide: flupyradifurone, organophosphate: fenitrothion, carbamate: carbaryl, formamidine: amitraz, and pyrethroid: bifenthrin) were purchased from Sigma-Aldrich (Merck, Saint Louis, MO, USA). The pesticides were dissolved in 100% acetone and stored at -20°C until use. To optimize the concentration of pesticides, we preliminarily treated the honey bees with the recommended field concentration of each pesticide; however, when honey bees showed high mortality at the field concentration, the concentration was reduced until it reached approximately LD_20_. The pesticide concentrations optimized in this study were: 40 ppm for acetamiprid; 1 ppm for imidacloprid; 85.5 ppm for flupyradifurone; 7.5 ppm for fenitrothion; 7.5 ppm for carbaryl; 200 ppm for amitraz; and 20 ppm for bifenthrin. Based on the test concentration of each pesticide, stock solution (×100) was prepared with 100% acetone, and then diluted 100-fold with 50% (weight/volume) sucrose solution. Ten microliters of the pesticide solution were orally administered to the honey bees. Honey bees were anesthetized with ice and fixed between the abdomen and thorax using stapler pins on a hard Styrofoam plate. Pesticide-treated honey bees were transferred to plastic cups containing 50% sucrose solution and maintained at 28°C and 60% relative humidity. As pesticide-treated samples were prepared in previous studies [49], at 24 h after pesticide exposure, the surviving honey bees were dissected into the head, thorax, gut, fat body, and carcass (excluding gut and fat body from the abdomen) under a microscope (Olympus, Tokyo, Japan).

### Total RNA extraction, primer design, and qRT-PCR

RNA was extracted from prepared body parts (three replicates; five bees/replication) using the yesR™ total RNA extraction kit with a gDNA Eliminator column (Prefilter PF02) (GenesGen, Busan, Korea). The purity and quantity of the extracted RNA were measured in triplicate using a SpectraMax QuickDrop spectrophotometer (Molecular Devices, CA, USA). For cDNA synthesis, 1 μg of total RNA was primed with oligo (dT), and cDNA was synthesized using ReverTra Ace reverse transcriptase, according to the manufacturer’s protocol (Toyobo, Osaka, Japan). The CFX Connect Real-Time PCR Detection System (Bio-Rad, Hercules, CA, USA) was used for qRT-PCR with SYBR GREEN methodology.

Primers of candidate reference and target genes were designed according to previous studies [28, 30, 32, 50], and those for *AChE1* were newly designed in this study using Oligo Calc software (http://biotools.nubic.northwestern.edu/OligoCalc.html) based on sequence information (GenBank accession no. XM_016914793) (S1 Table).

The PCR efficiency of each primer set was calculated from the given slope after running a standard curve generated with three points of 10-fold serial dilutions of cDNA using the E = 10^−1/slope^ formula. qRT-PCR was conducted in duplicate (technical replicates) for a final volume of 20 μL, containing 10 pmol of each primer, 2x Thunderbird SYBR qPCR Master Mix (Toyobo, Osaka, Japan), and 5 μL of synthesized cDNA. The PCR was performed using the following protocol: 95°C for 1 min; and then 40 cycles at 95°C for 15 s, 56°C for 15 s, and 72°C for 30 s. One cycle of melting curve analysis (65°C to 95°C in 0.5°C increments) was carried out to check the amplicon specificity. The quantification cycle (C_q_) values of five candidate reference genes and target genes (*AChE1*, *CYP9Q1*, *CYP9Q2*, *CYP9Q3*, *SOD1*, and *CAT*) were obtained from the different body parts of honey bees treated with the seven pesticides and control bees (no pesticide treatment) at the same fluorescence threshold line (0.1).

### Ranking the expression stabilities of candidate reference genes

**Fig 1:**
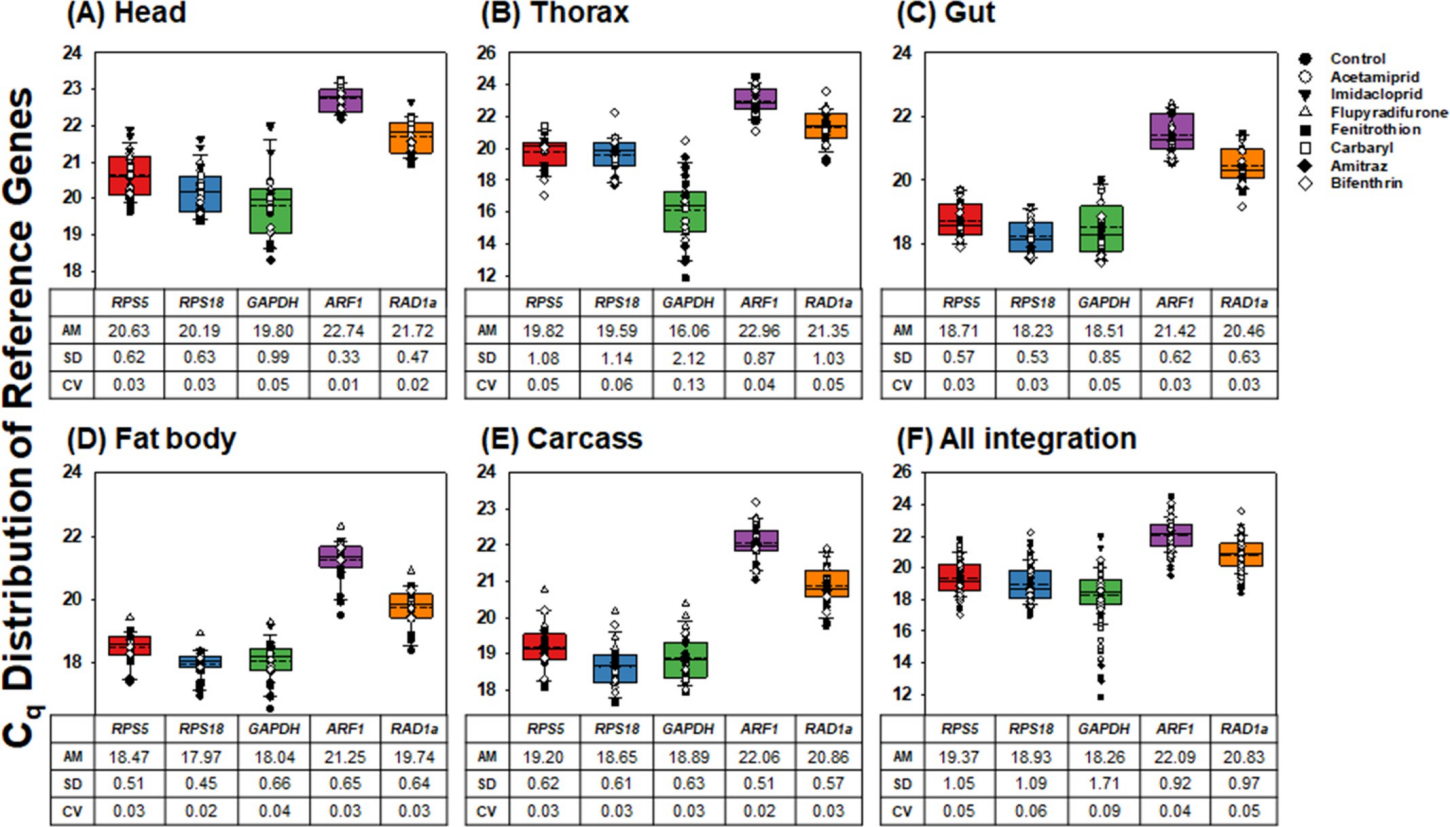
C_q_ distributions of the five candidate reference genes. Box plots of C_q_ values for the five reference genes were compared from five body parts from honey bees exposed to seven pesticides (A-E), and the integration of all samples (F). The horizontal lines in the box indicate the 25^th^, 50^th^, and 75^th^ percentile values. The dotted lines in the box show the mean median. The error bars denote the maximum and minimum values.

**Fig 2:**
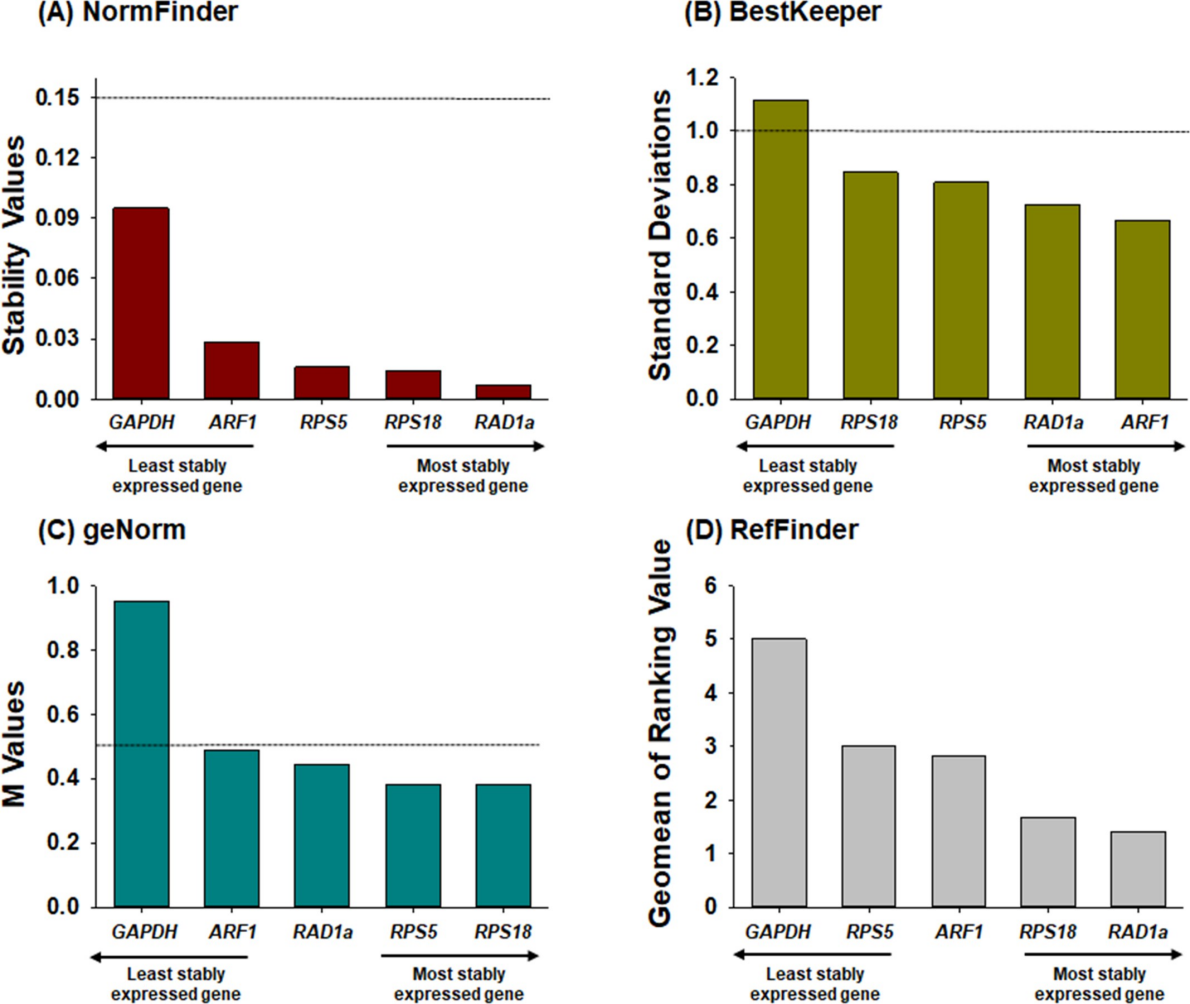
The expression stability values of the five candidate reference genes analyzed by four programs. The C_q_ values integrated from all five body parts of honey bees exposed to seven pesticides were used for the analysis with NormFinder (A), BestKeeper (B), geNorm (C), and RefFinder (D). The dotted line indicates the cutoff values for the appropriate reference gene selection.

**Fig 3:**
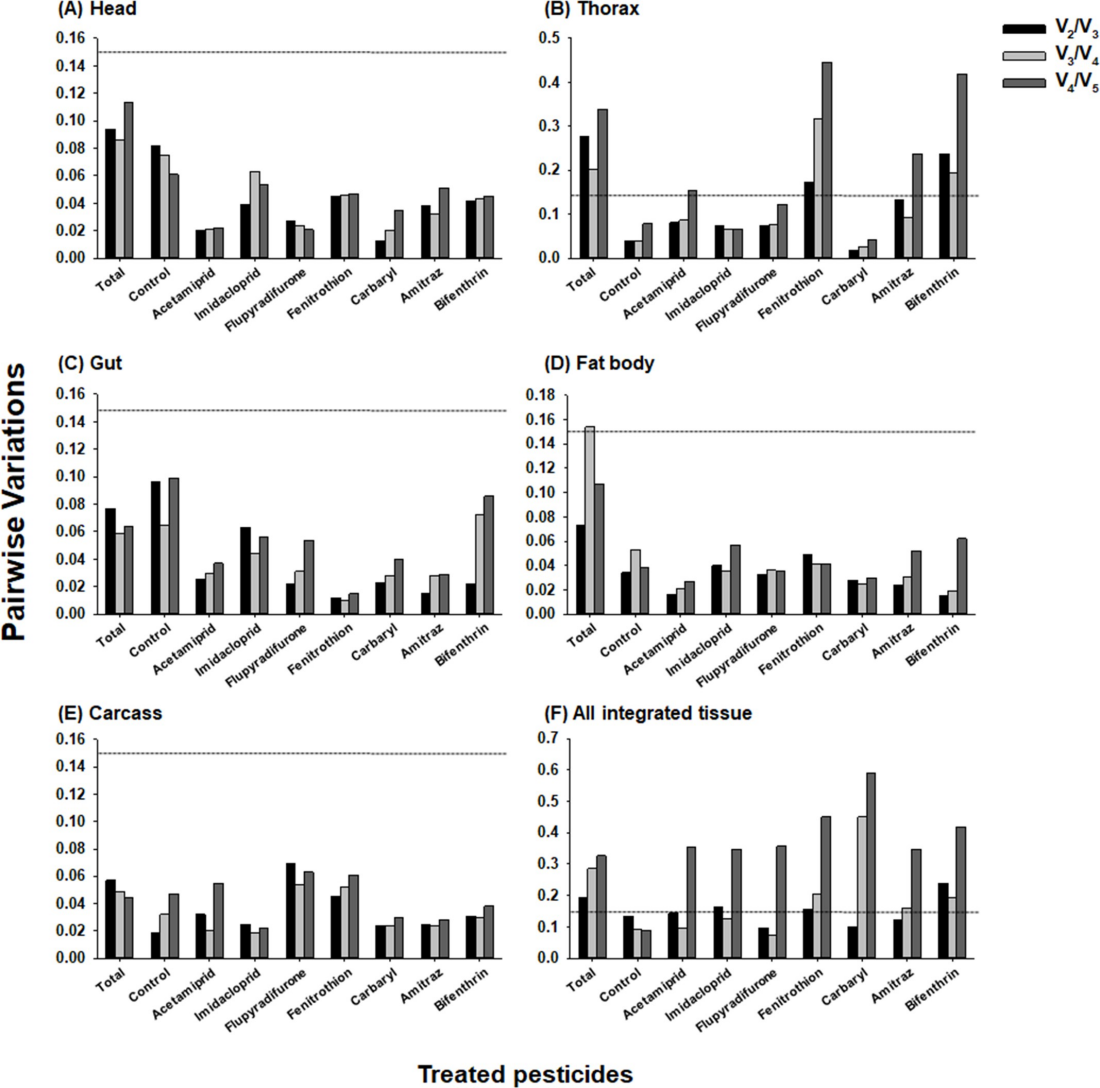
Optimal number of reference genes for target gene normalization determined by geNorm pairwise variation analysis. Pairwise variation values (V_n_/V_n+1_) were calculated from the five body parts of honey bees treated with seven pesticides (A-E), and integration of all samples (F). The dotted lines indicate the cutoff value for the suggestion of an optimal number of reference genes.

The C_q_ distribution of candidate reference genes across the seven types of pesticides and five body parts was analyzed using SigmaPlot 14.0, and the arithmetic mean (AM), standard deviation (SD), and coefficient of variation (CV) values were calculated (CV = SD/AM) (Fig 1). To analyze the expression stability of the five reference genes across different body parts and different pesticide treatment conditions, four programs (Table 1 and Fig 2): geNorm (version 3.3) [51], NormFinder (version 0.953) [52], BestKeeper (version 1) [53], and RefFinder [54] were used. NormFinder automatically calculates the stability values for all candidate reference gene-based overall variations to evaluate the systematic error introduced for gene normalization. Lower stability values indicate more stable genes; therefore, NormFinder ranked all candidates according to their stability values (S2 Table). BestKeeper identifies suitable reference genes based on the geometric mean of the C_q_ and SD values. The more stable genes have lower SD values, which are used in selecting a suitable reference gene [53] (S3 Table). The geNorm program calculates the expression stability M value, with more stable genes having lower M values (S4 Table). geNorm also reveals the analysis results of pairwise variation (V_n_/V_n+1_) to suggest the optimal number of references for the normalization of target genes (Fig 3). RefFinder provides a comprehensive ranking by combining the values of the three previous programs and the comparative Delta-C_q_ method [54] (S5 Table).

**Table 1:** Comprehensive ranking of reference genes calculated using NormFinder, BestKeeper, geNorm, and RefFinder in different body parts under different pesticide treatment conditions.

Body parts and Pesticide	Rank	Program			
		NormFinder^a^	BestKeeper^b^	geNorm^c^	RefFinder^d^
**Head**	1	*RPS5* (0.003)	*ARF1* (0.291)	*RPS5* (0.206)	*RPS5* (1.141)
	2	*RPS18* (0.003)	*RAD1a* (0.407)	*RPS18* (0.213)	*RPS18* (1.861)
	3	*RAD1a* (0.009)	*RPS18* (0.512)	*RAD1a* (0.238)	*RAD1a* (2.711)
	4	*ARF1* (0.021)	*RPS5* (0.536)	*ARF1* (0.298)	*ARF1* (2.282)
	5	*GAPDH* (0.028)	*GAPDH* (0.765)	*GAPDH* (0.410)	*GAPDH* (5.000)
**Thorax**	1	*RPS5* (0.013)	*ARF1* (0.706)	*RPS5* (0.583)*	*RPS18* (1.414)
	2	*RPS18* (0.013)	*RAD1a* (0.724)	*RPS18* (0.589)*	*RAD1a* (1.682)
	3	*ARF1* (0.041)	*RPS5* (0.834)	*ARF1* (0.675)*	*ARF1* (2.280)
	4	*RAD1a* (0.056)	*RPS18* (1.037)*	*RAD1a* (0.790)*	*RPS5* (3.722)
	5	*GAPDH* (0.166)*	*GAPDH* (2.865)*	*GAPDH* (1.179)*	*GAPDH* (5.000)
**Gut**	1	*RPS5* (0.005)	*RPS18* (0.433)	*RPS5* (0.185)	*RPS5* (1.189)
	2	*RAD1a* (0.006)	*RPS5* (0.474)	*RPS18* (0.196)	*RPS18* (2.000)
	3	*ARF1* (0.010)	*RAD1a* (0.515)	*RAD1a* (0.211)	*RAD1a* (2.449)
	4	*RPS18* (0.011)	*ARF1* (0.533)	*ARF1* (0.237)	*ARF1* (3.464)
	5	*GAPDH* (0.018)	*GAPDH* (0.701)	*GAPDH* (0.282)	*GAPDH* (5.000)
**Fat body**	1	*RAD1a* (0.005)	*RPS18* (0.205)	*RPS5* (0.213)	*RPS5* (1.189)
	2	*ARF1* (0.007)	*RPS5* (0.284)	*RPS18* (0.213)	*RPS18* (2.000)
	3	*RPS5* (0.008)	*ARF1* (0.353)	*RAD1a* (0.222)	*RAD1a* (2.632)
	4	*RPS18* (0.013)	*RAD1a* (0.373)	*ARF1* (0.425)	*ARF1* (3.224)
	5	*GAPDH* (0.018)	*GAPDH* (0.447)	*GAPDH* (0.480)	*GAPDH* (5.000)
**Carcass**	1	*RAD1a* (0.005)	*ARF1* (0.379)	*RPS5* (0.153)	*RAD1a* (1.565)
	2	*RPS5* (0.005)	*RAD1a* (0.443)	*RPS18* (0.155)	*RPS5* (2.000)
	3	*RPS18* (0.007)	*RPS18* (0.453)	*RAD1a* (0.164)	*RPS18* (2.280)
	4	*GAPDH* (0.011)	*RPS5* (0.455)	*ARF1* (0.188)	*ARF1* (2.828)
	5	*ARF1* (0.011)	*GAPDH* (0.490)	*GAPDH* (0.211)	*GAPDH* (5.000)
**Acetamiprid**	1	*RAD1a* (0.007)	*ARF1* (0.448)	*RPS5* (0.321)	*RAD1a* (1.316)
	2	*ARF1* (0.017)	*RAD1a* (0.571)	*RPS18* (0.337)	*ARF1* (2.213)
	3	*RPS5* (0.017)	*RPS5* (0.770)	*RAD1a* (0.372)	*RPS18* (2.632)
	4	*RPS18* (0.024)	*RPS18* (0.881)	*ARF1* (0.400)	*RPS5* (2.632)
	5	*GAPDH* (0.102)	*GAPDH* (1.200)*	*GAPDH* (0.954)*	*GAPDH* (5.000)
**Imidacloprid**	1	*RPS5* (0.006)	*ARF1* (0.496)	*RPS5* (0.320)	*RPS18* (1.414)
	2	*RAD1a* (0.007)	*RAD1a* (0.655)	*RPS18* (0.351)	*RPS5* (1.861)
	3	*RPS18* (0.027)	*RPS5* (0.879)	*RAD1a* (0.399)	*RAD1a* (2.711)
	4	*ARF1* (0.028)	*RPS18* (0.992)	*ARF1* (0.465)	*ARF1* (2.828)
	5	*GAPDH* (0.096)	*GAPDH* (1.457)*	*GAPDH* (0.984)*	*GAPDH* (5.000)
**Flupyradifurone**	1	*RAD1a* (0.007)	*ARF1* (0.574)	*RPS18* (0.219)	*RAD1a* (1.316)
	2	*RPS18* (0.010)	*RAD1a* (0.626)	*RPS5* (0.226)	*ARF1* (1.565)
	3	*RPS5* (0.012)	*RPS5* (0.747)	*RAD1a* (0.250)	*RPS5* (2.828)
	4	*ARF1* (0.018)	*RPS18* (0.762)	*ARF1* (0.289)	*RPS18* (3.464)
	5	*GAPDH* (0.099)	*GAPDH* (1.272)*	*GAPDH* (0.891)*	*GAPDH* (5.000)
**Fenitrothion**	1	*RPS5* (0.006)	*RAD1a* (0.568)	*RPS5* (0.372)	*ARF1* (1.732)
	2	*RPS18* (0.006)	*RPS5* (0.643)	*RPS18* (0.382)	*RPS5* (1.861)
	3	*RAD1a* (0.031)	*RPS18* (0.690)	*ARF1* (0.444)	*RPS18* (1.861)
	4	*ARF1* (0.036)	*ARF1* (0.716)	*RAD1a* (0.544)*	*GAPDH* (3.364)
	5	*GAPDH* (0.141)	*GAPDH* (1.354)*	*GAPDH* (1.201)*	*RAD1a* (5.000)
**Carbaryl**	1	*ARF1* (0.006)	*ARF1* (0.781)	*RPS18* (0.221)	*RPS18* (1.000)
	2	*RAD1a* (0.006)	*RAD1a* (0.784)	*RPS5* (0.242)	*RPS5* (2.213)
	3	*RPS18* (0.012)	*RPS18* (0.946)	*RAD1a* (0.257)	*ARF1* (2.280)
	4	*RPS5* (0.026)	*GAPDH* (0.961)	*ARF1* (0.275)	*RAD1a* (4.229)
	5	*GAPDH* (0.097)	*RPS5* (1.048)*	*GAPDH* (0.872)*	*GAPDH* (4.729)
**Amitraz**	1	*RPS18* (0.006)	*RPS5* (0.620)	*RPS18* (0.281)	*RPS5* (1.189)
	2	*RAD1a* (0.009)	*RPS18* (0.652)	*RPS5* (0.303)	*ARF1* (1.861)
	3	*RPS5* (0.012)	*RAD1a* (0.697)	*RAD1a* (0.330)	*RPS18* (2.449)
	4	*ARF1* (0.047)	*ARF1* (0.718)	*ARF1* (0.494)	*GAPDH* (4.162)
	5	*GAPDH* (0.108)	*GAPDH* (1.193)*	*GAPDH* (0.994)*	*RAD1a* (4.472)
**Bifenthrin**	1	*ARF1* (0.021)	*RPS5* (0.874)	*RPS5* (0.478)	*RAD1a* (1.565)
	2	*RPS5* (0.023)	*ARF1* (0.907)	*RPS18* (0.482)	*RPS18* (1.861)
	3	*RAD1a* (0.026)	*GAPDH* (0.963)	*ARF1* (0.604)*	*RPS5* (2.449)
	4	*RPS18* (0.031)	*RAD1a* (0.984)	*RAD1a* (0.686)*	*ARF1* (3.344)
	5	*GAPDH* (0.066)	*RPS18* (1.125)*	*GAPDH* (0.837)*	*GAPDH* (4.229)
**Control**	1	*RPS5* (0.008)	*GAPDH* (0.815)	*RPS5* (0.253)	*RPS5* (1.414)
	2	*RAD1a* (0.010)	*ARF1* (0.915)	*RPS18* (0.277)	*RPS18* (1.861)
	3	*RPS18* (0.018)	*RAD1a* (0.939)	*RAD1a* (0.320)	*RAD1a* (2.711)
	4	*ARF1* (0.018)	*RPS5* (0.981)	*ARF1* (0.360)	*ARF1* (2.828)
	5	*GAPDH* (0.021)	*RPS18* (1.038)*	*GAPDH* (0.417)	*GAPDH* (5.000)
**All**	1	*RAD1a* (0.007)	*ARF1* (0.665)	*RPS18* (0.380)	*RAD1a* (1.414)
	2	*RPS18* (0.014)	*RAD1a* (0.725)	*RPS5* (0.383)	*RPS18* (1.682)
	3	*RPS5* (0.016)	*RPS5* (0.810)	*RAD1a* (0.445)	*ARF1* (2.828)
	4	*ARF1* (0.028)	*RPS18* (0.845)	*ARF1* (0.487)	*RPS5* (3.000)
	5	*GAPDH* (0.095)	*GAPDH* (1.116)*	*GAPDH* (0.952) *	*GAPDH* (5.000)

Note: The stabilities of five reference genes were measured in each body part treated with seven pesticides and each pesticide-treated sample was integrated from five body parts.

^a^ Stability value

^b^ Standard deviation

^c^ Average expression stability M

^d^ Geomean of ranking value

* Stability values exceeded the cut-off value

### Validation of reference genes by normalization of *AChE1* expression levels

**Fig 4:**
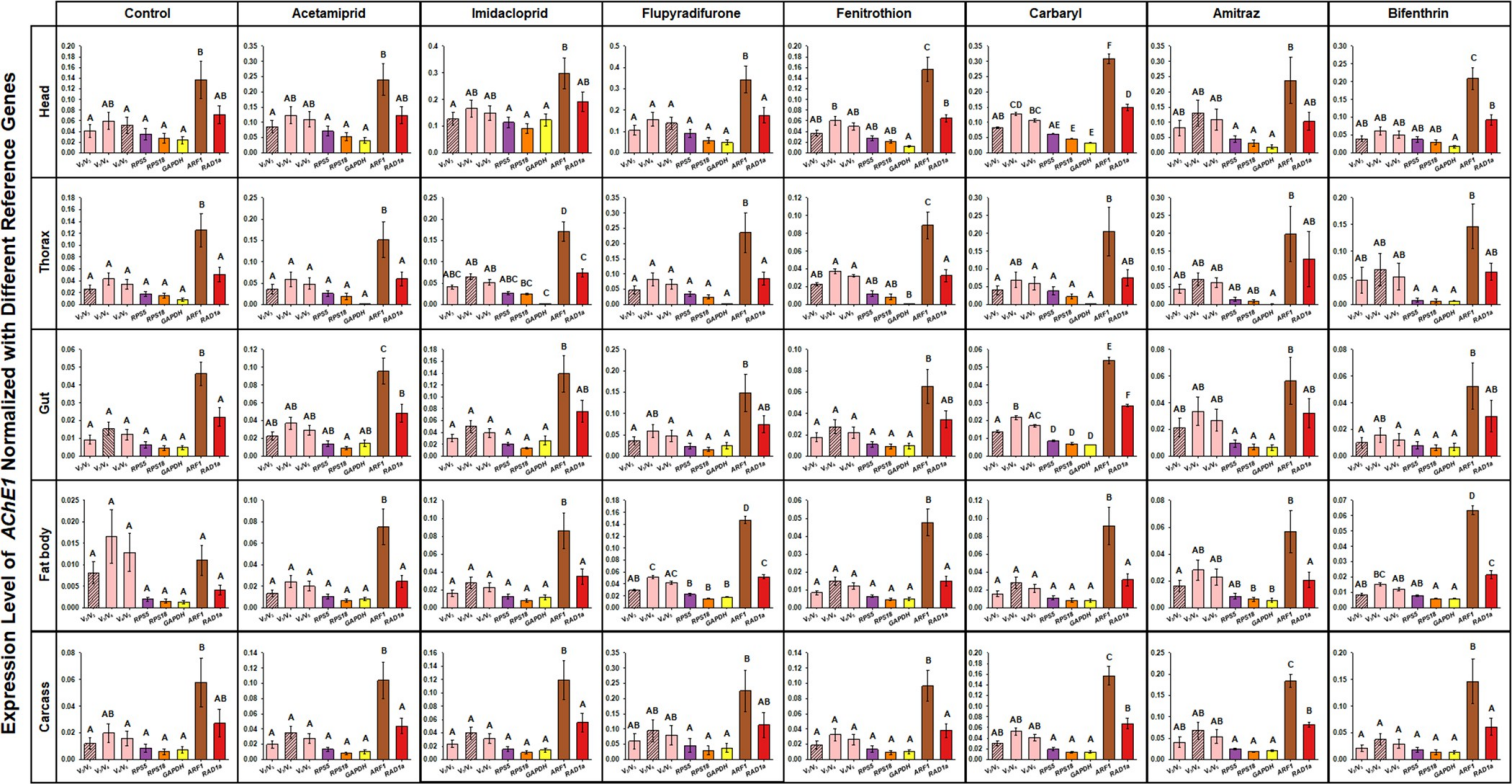
The comparison of expression levels of *AChE1* calculated with different normalization methods. The expression levels of *AChE1* normalized with a single gene of the five candidate reference genes and combinations of multiple reference genes were statistically compared in the five body parts of honey bees exposed to seven pesticides and non-pesticide treatment control. The reference genes were selected according to the stability ranks analyzed by RefFinder: combinations of two (*RAD1a+RPS18*), three (*RAD1a+RPS18+ARF1*), and four (*RAD1a+RPS18+ARF1+RPS5*) (Fig 2D). The expression levels of *AChE1* calculated with different normalization methods were statistically compared with one-way ANOVA with Tukey’s multiple comparison test, and different letters indicate significantly different values (*p* < 0.05). The hatched lines indicate the expression levels of *AChE1* normalized with the combination of multiple reference genes of which the number was obtained from the lowest Pairwise variation values (V_n_/V_n+1_) (Fig 3). Data are presented as mean ± SE.

The C_q_ values from the reference genes and *AChE1* were obtained from the same sample of body parts, and the expression level of *AChE1* was normalized by the relative quantification method modified from the original concept of 2^-ΔΔCq^ [55]. To select common reference gene(s) for target gene normalization across different sample conditions, the expression levels of *AChE1* normalized to one of five genes and combinations of multiple reference genes were statistically compared in the different body parts of honey bees treated with seven pesticides using SPSS for Windows (version 23.0) with one-way analysis of variance (ANOVA) test and Tukey’s multiple comparison test (Fig 4). To apply multiple reference genes to normalize *AChE1* expression levels, reference genes were selected based on the rank of the stable gene analyzed by RefFinder: combinations of two (*RAD1a+RPS18*), three (*RAD1a+RPS18+ARF1*), and four (*RAD1a+RPS18+ARF1+RPS5*) (Fig 2D),

### Expression profiles of detoxification-related genes

**Fig 5:**
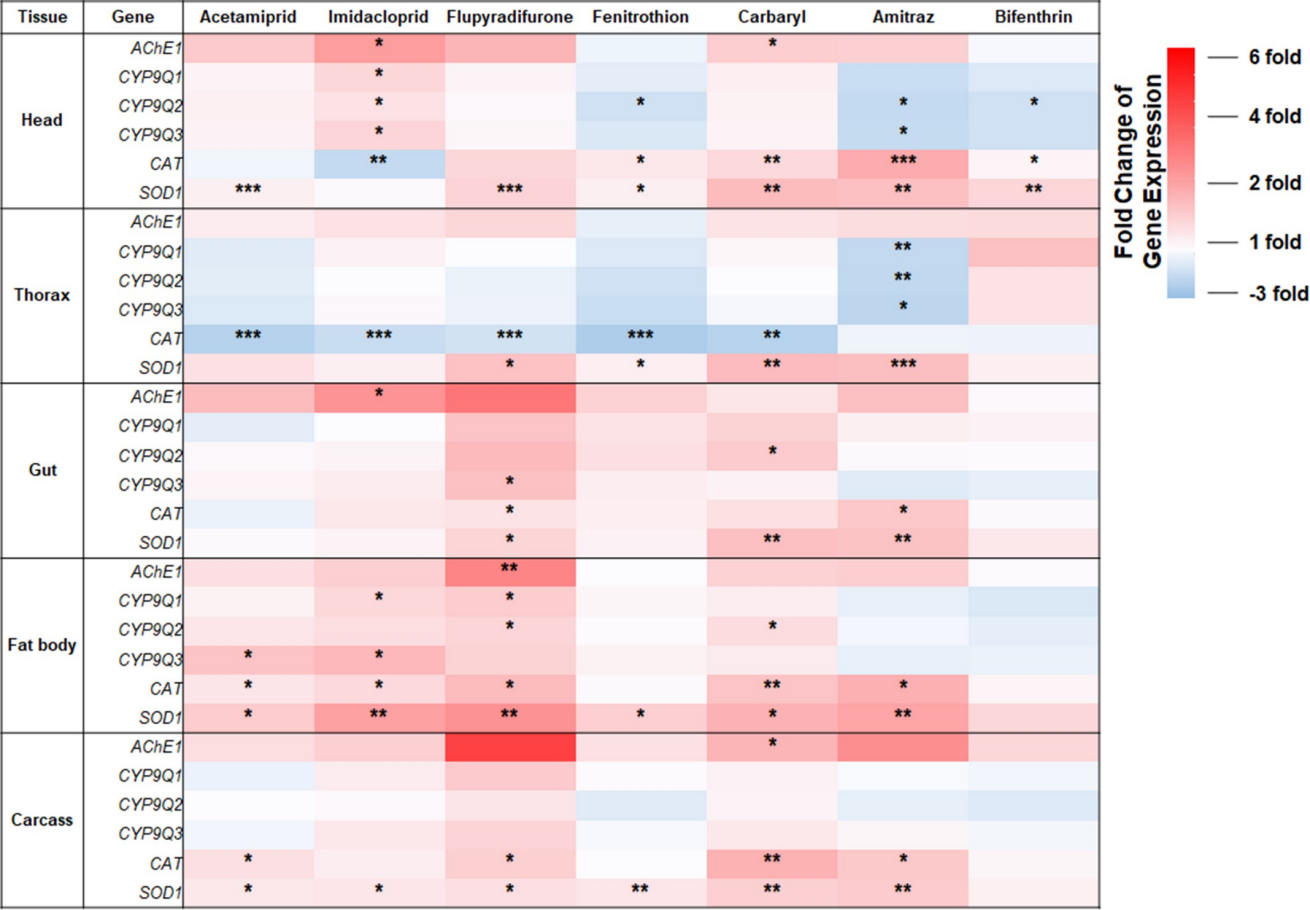
Expression levels of *AChE1*, *CYP9Q1-3*, *CAT*, and *SOD1* in honey bee body parts treated with pesticides. The expression levels of genes were normalized with a combination of *RAD1a* and *RPS18*, and the expression changes of each gene due to pesticide exposure were calculated with the non-pesticide treatment control. The difference of expression between the control and pesticide-treated sample were statistically compared with the independent samples T-test with Tukey’s comparison analysis, and significant differences were measured with *(*p* < 0.05), **(*p* < 0.01), and ***(*p* < 0.001). Red presents upregulation, while blue presents downregulation.

To investigate the expression of target genes (*AChE1*, *CYP9Q1*, *CYP9Q2*, *CYP9Q3*, *SOD1*, and *CAT*) possibly induced by pesticide treatment in five different body parts, their expression levels normalized to the combination of the two most stable genes, *RAD1a* and *RPS18*, were statistically compared between the control (no pesticide treatment) and pesticide treatment conditions using the independent samples T-test with Tukey’s comparison analysis (Figs 5 and S2–S6).

## Results

### Amplification efficiency and C_q_ distribution analysis

Amplification specificities and efficiencies of the five candidate reference genes and target genes were examined using the primer sets used in this study. All PCR products showed a single band upon electrophoresis and a clear single peak in the melting curve analysis with real-time PCR (S1 Fig). As observed in previous studies [28, 30, 32, 50], eleven genes had linear regression coefficients R^2^ > 0.994 and efficiencies of 92–107%, indicating that all primer sets used in this study were acceptable (S1 Table).

The expression levels of the five putative reference genes in the five body parts were analyzed (Fig 1). Based on the obtained C_q_ value for each gene, AM, SD, and CV values were determined. Based on the CV values, *ARF1* and *GAPDH* were determined to be the most stable and least stable genes, respectively, in the head, thorax, and gut (Fig 1A–1C). In the fat body, the CV value of *GAPDH* (0.04) was higher than that of the other four genes, whereas *RPS18* exhibited the lowest CV value (0.02). The CV values of the other three genes (*RPS5*, *ARF1*, and *RAD1a*) were 0.03 (Fig 1D). *ARF1* showed the lowest CV value (0.02), whereas the CV values of the other four genes (*RPS5*, *RPS18*, *GAPDH*, and *RAD1a*) were equal (0.03) in the carcass (Fig 1E). The integrated CV values were calculated after the C_q_ values of each gene from the five different body parts from the honey bees exposed to the seven pesticides and those from the control group (no pesticide treatment) were combined. As judged by all integrated CV values, the stability rank from the most stable (lowest CV value) to the least (highest value) was as follows: *ARF1* (0.04) > *RPS5* = *RAD1a* (0.05) > *RPS18* (0.06) > *GAPDH* (0.09) (Fig 1F). Considering that a CV < 1 indicates low variance [56], all five genes are acceptable as reference genes for the different body parts from honey bees exposed to various pesticides.

### Ranking of expression stability from the four programs

#### NormFinder

Although stability values analyzed by NormFinder for each gene were varied across different pesticide treatment conditions and body parts (S2 Table), *RPS5* was ranked as the most stable gene in the head, thorax, and gut, whereas it was the third most stable with *RAD1a* being the most stable gene in the fat body and carcass (S2 Table). When the expression variations of each gene obtained from the five body parts were combined, *RAD1a* was ranked as the most stable gene under acetamiprid, flupyradifurone, and carbaryl exposure, and *RPS5* was the optimal gene in the control, imidacloprid, and fenitrothion treatment conditions. In addition, *ARF1* was most stably expressed in carbaryl- and bifenthrin-treated bees. In the amitraz treatment group, *RPS18* was the most stable (Table 1). When all expression stability values were integrated from the five body parts, the stability rank from the most to least stable was as follows: *RAD1a* > *RPS18* > *RPS5* > *ARF1* >> *GAPDH* (Table 1 and Fig 2A). Considering a stability value < 0.15 as the criterion for suitable reference gene selection in NormFinder [57, 58], all genes were acceptable across different conditions, but *GAPDH* (stability value = 0.166) was not suggested as a qRT-PCR reference gene for thorax of honey bees exposed to pesticides (Table 1 and Fig 2A).

#### BestKeeper

Candidate genes with low SD (usually < 1.0) are suggested as appropriate reference genes by the BestKeeper analysis [59]. The SD values of candidate reference genes varied across different body parts of honey bees treated with various pesticides (S3 Table). As indicated by SD values < 1.0, all five genes were determined to be optimal reference genes in the head, gut, fat body, and carcass of honey bees exposed to the seven pesticides, and the control. In the thorax, *ARF1*, *RAD1a*, and *RPS5* exhibited SD values < 1.0, and the SD of *RPS18* (1.037) was slightly higher than 1.0, whereas that of *GAPDH* was 2.865 (Table 1). When data from the different body parts treated with acetamiprid, imidacloprid, flupyradifurone, fenitrothion, and amitraz were integrated, *GAPDH* was found to be unstable for target gene normalization due to its high SD values (> 1.0). In the case of carbaryl and bifenthrin treatments, the respective SD values of *RPS5* and *RPS18* were slightly higher than 1 (Table 1). In the control group, *RPS18* exhibited a slightly higher SD value; however, the other four genes were applicable as appropriate reference genes. Based on the SD values of all integrated data, four genes (*ARF1*, *RAD1a*, *RPS5*, and *RPS18*) were determined as optimal reference genes, but the SD value of *GAPDH* was slightly higher than the cut-off line (1.116) (Table 1 and Fig 2B).

#### geNorm

The average expression stability values (M values) were analyzed using geNorm across the body parts of honey bees exposed to different pesticides (S4 Table). As suggested in previous studies, an M value under 0.5 (M ≤ 0.5) is an acceptable criterion for the selection of reference genes [60, 61]. In each sample of body parts, the M values of all five candidate reference genes were below the cut-off value (0.5), except for the thorax sample. In the thorax, the M values of *RPS5* (0.583) and *RPS18* (0.589) were slightly higher than the cut-off value (0.5), whereas those of *ARF1*, *RAD1a*, and *GAPDH* were 0.675, 0.79, and 1.179, respectively (Table 1). According to the M values integrated from all body parts treated with each pesticide, *RPS5* and *RPS18* were determined to be stably expressed in each pesticide treatment, while the M values of *RAD1a* were higher than 0.5 in fenitrothion and bifenthrin treatment conditions, and that of *ARF1* was also higher than the cut-off value in the bifenthrin sample. In addition, *GAPDH* was not recommended as an optimal reference gene for all pesticide treatments (Table 1). In all integrated analyses, the M values of four genes (*RPS18*, *RPS5*, *RAD1a*, and *ARF1*) were < 0.5, whereas that of *GAPDH* was 0.952 (Table 1 and Fig 1C).

In addition to the M value analysis, the number of reference genes for reliable normalization of target genes in qRT-PCR was determined using pairwise variation (V_n_/V_n+1_) values calculated in geNorm (Fig 3). Based on the cutoff value (0.15) suggested in previous studies [61, 62], any number of reference combinations was applicable for target gene normalization in the head, gut, fat body, and carcass because all subgroups showed lower pairwise variations (V_2_/V_3_, V_3_/V_4_, and V_4_/V_5_) than the cutoff value (0.15) (Fig 3A, 3C, 3D and 3E). However, in the thorax, V_n_/V_n+1_ values varied depending on the pesticide (Fig 3B). In the thorax, the lowest V_n_/V_n+1_ values in the fenitrothion and bifenthrin treatments were V_2_/V_3_ (0.173) and V_3_/V_4_ (0.193), and the combined pesticide treatment data showed the lowest V_n_/V_n+1_ values at V_3_/V_4_ (0.202) (Fig 3B). When all data from different body parts treated with pesticides were integrated, V_n_/V_n+1_ values lower than the cutoff value were obtained for the control and five pesticide treatment groups (acetamiprid, imidacloprid, flupyradifurone, carbaryl, and amitraz) (Fig 3F). The V_2_/V_3_ ratio was 0.157 in the fenitrothion treatment, and V_3_/V_4_ was 0.193 in the bifenthrin treatment group. The lowest V_n_/V_n+1_ value was 0.192 at V_2_/V_3_ for all integrated data (Fig 3F).

#### RefFinder

Based on the three different algorithms, comprehensive stability was calculated, and the ranks of candidate reference genes were obtained using RefFinder (Tables 1 and S5). The comprehensive rank of each gene varied depending on the body parts and pesticide treatment (S5 Table). In general, *RPS5* was ranked as the most stable gene in the head, gut, and fat body, but it was the second and fourth most stable gene in the carcass and thorax, respectively. *RPS18* was ranked first in the thorax but the second most stable gene in the head, gut, and fat body. In the carcass, *RAD1a* was ranked as the most stable gene (Table 1). When body part samples were combined from each pesticide treatment condition, *RAD1a* was the most stable gene in the acetamiprid, flupyradifurone, and bifenthrin treatments. *RPS18* was ranked as the most stable gene under imidacloprid and carbaryl exposure conditions, whereas *RPS5* was determined to be the most stable gene under amitraz treatment conditions and in the control group body parts. In the fenitrothion treatment, *ARF1* was selected as the most stable gene (Table 1). When the comprehensive rank was calculated from the integrated data of the five body parts treated with seven pesticides, the stability rank from the most to least stable was as follows: *RAD1a* > *RPS18* > *ARF1* > *RPS5* > *GAPDH* (Table 1 and Fig 2D).

### Validation of reference genes for target gene normalization

According to the pairwise variation values analyzed by geNorm, the optimal number of reference genes for target gene normalization varied depending on the body parts, pesticide treatment, and the control (Fig 3). We selected *AChE1* as the target gene for the validation of candidate reference genes by comparing its expression levels normalized by a single gene among five candidates and combinations of different numbers of reference genes. Based on the rank of the stable gene in the RefFinder analysis (Fig 2D), candidate reference genes were selected for the combinations of multiple reference genes. When expression levels of *AChE1* normalized with the combination of two (*RAD1a+RPS18*), three (*RAD1a+RPS18+ARF1*), and four (*RAD1a+RPS18+ARF1+RPS5*) reference genes were compared, in general, they exhibited statistically similar transcription levels of *AChE1* except for the carbaryl-treated head, carbaryl-treated gut, and flupyradifurone-treated fat body (Fig 4). However, in these conditions, the combination of two genes was suggested by pairwise variation analysis (Fig 3). These indicate that the combination of two genes (*RAD1a+RPS18*) is sufficient for target gene normalization rather than three or four genes.

When the expression levels of *AChE1* were normalized with a single gene among the five genes, those normalized with *RPS5*, *RPS18*, and *GAPDH* showed significantly similar expression levels in all body parts and pesticide treatment groups (Fig 4). However, if the expression levels of *AChE1* normalized with one of these three single genes were compared with those calculated with the combination of two reference genes, they computed statistically different expression levels of *AChE1* in carbaryl-treated head and gut, suggesting that application of a single gene is not appropriate for comparing target gene expression levels across various body parts of honey bees treated with different pesticides.

### Expression profiles of genes putatively involved in the detoxification mechanism

As a combination of two genes (*RAD1a+RPS18*) was suggested to be the most suitable method for target gene normalization (Fig 4), the expression levels of genes putatively involved in detoxification mechanisms were normalized with the combination of *RAD1a* and *RPS18* (Figs 5 and S2–S6). As summarized in Fig 5, the induction or reduction ratio of each gene did not show significant trends depending on the body parts and pesticide when the expression ratio of genes after pesticide treatment was compared with that of the non-treated control. However, expression of genes in the gut, fat body, and carcass, as compared to that in the head and thorax, were generally induced by pesticides. Among the seven pesticides, in the head, imidacloprid significantly induced the expression of *AChE1* and three *CYP9Qs* (*p* < 0.05) and significantly reduced *CAT* expression (*p* < 0.01). However, *CAT* was significantly induced by exposure to fenitrothion, carbaryl, amitraz, and bifenthrin (*p* < 0.05), whereas the expression levels of *SOD1* were significantly higher than those in the control group when honey bees were treated with acetamiprid, flupyradifurone, fenitrothion, carbaryl, amitraz, and bifenthrin (Fig 5, see head; S2 Fig). In the thorax, amitraz reduced the expression of *CYP9Qs* (*p* < 0.05). Interestingly, *CAT* expression was significantly reduced by exposure to five pesticides (acetamiprid, imidacloprid, flupyradifurone, fenitrothion, and carbaryl), whereas *SOD1* expression was induced by four pesticides (flupyradifurone, fenitrothion, carbaryl, and amitraz) (*p* < 0.05) in the thorax (Fig 5, see thorax; S3 Fig). In the case of the gut, *AChE1*, *CYP9Q2*, and *CYP9Q3* were induced by imidacloprid, carbaryl, and flupyradifurone, respectively, whereas *CAT* was induced by flupyradifurone and amitraz (*p* < 0.05). Three pesticides (flupyradifurone, carbaryl, and amitraz) significantly induced the expression of *SOD1* (*p* < 0.05) in the gut (Fig 5, see gut; S4 Fig). In the fat bodies, six and five pesticides strongly induced the expression of *SOD1* and *CAT* (*p* < 0.05), respectively, whereas *AChE1* was induced by flupyradifurone, and three *CYP9Qs* were induced by respective two pesticides (*p* < 0.05). Among the pesticides, flupyradifurone strongly induced the expression of five genes (*AChE1*, *CYP9Q1*, *CYP9Q2*, *CAT*, and *SOD1*) (*p* < 0.05) (Fig 5, see fat body; S5 Fig). In the carcasses of pesticide-treated honey bees, the expression levels of *AChE1* and three *CYP9Qs* did not differ from those of the control. Four and five pesticides significantly affected the expression levels of *CAT* and *SOD1*, *respectively* (*p* < 0.05) (Fig 5, see carcass; S6 Fig).

## Discussion

### Selection of optimal reference gene for qRT-PCR in pesticide-exposed honey bee body parts

Since, in addition to foragers, nurse bees have been suggested to also be possibly exposed to agricultural chemicals via pesticide-contaminated nectar and pollen [18–22], accurate determination of gene expression levels is essential to identify the genes putatively involved in the physiological response to pesticides in nurse bees; the expression profiles significantly induced or reduced by pesticide exposure can be used as molecular markers to identify the pesticide damaging honey bee colonies. Owing to the possibility of variability in reference gene expression, the selection of appropriate reference genes that are stably expressed across different pesticide treatment conditions is necessary before conducting qRT-PCR for accurate determination of target gene expression. In the previous study [32], the optimal reference genes have been suggested in three body parts (head, thorax, and abdomen) of honey bees treated with four pesticides (imidacloprid, flupyradifurone, coumaphos, and fluvalinate). Considering that the detoxification mechanisms in various body parts may differ depending on the type of pesticide, in addition to three body parts (head, thorax, and abdomen), qRT-PCR reference genes should be evaluated in specific body parts, such as the gut and fat body, of pesticide-treated honey bees. Furthermore, various pesticides may affect honey bee colonies, therefore, expression stabilities of candidate reference genes should be additionally investigated in honey bees exposed to more various pesticides. Therefore, in this study, using pesticides and body parts that have not been previously studied, we evaluated the expression stabilities of five candidate reference genes using C_q_ distribution analysis and four different software platforms in honey bees.

Consistent with previous studies [28, 30, 63–65], the four programs resulted in different gene stability ranks depending on the different sample conditions (Table 1 and Fig 2 and S2–S5 Tables). geNorm analyzed pairwise variation as a guide to suggest the optimal number of reference genes for normalization of target gene expression (Fig 3). V_n_/V_n+1_ < 0.15 is usually used as a cutoff value in geNorm pairwise variation analysis [51, 61, 62]; however, the V_n_/V_n+1_ value seems to be applied leniently. According to previous studies [51, 66], V_n_/V_n+1_ < 0.2 was also suggested to be the cutoff value in pairwise variation analysis, and the lowest V_n_/V_n+1_ was widely applied to suggest the number of reference genes for target gene normalization regardless of the value [67], suggesting that the cutoff value of V_n_/V_n+1_ is not crucial. Based on the V_n_/V_n+1_ values in this study, geNorm pairwise variation analysis showed that combinations with different numbers of reference genes were suggested for target gene normalization in different conditions of honey bee samples, across body parts and pesticide treatments (Fig 3). However, to compare target gene expression levels across different body parts from honey bees exposed to various pesticides, selection of reference gene(s), which are commonly applied across different conditions, is essential. Therefore, to select common reference genes, we compared the expression levels of *AChE1* as a target gene, normalized with different combinations of multiple reference genes and a single gene among five candidate references in the five body parts treated with the seven pesticides (Fig 4). Since RefFinder provides a comprehensive ranking by combining the values of three programs (NormFinder, BestKeeper, and geNorm) and the comparative Delta-C_q_ method [54], references for multiple gene combinations were selected according to the integrated rank of RefFinder (Fig 2D). Although combinations of different numbers of genes were suggested by the geNorm pairwise variation analysis depending on different sample conditions (Fig 3), *AChE1* exhibited identical expression levels in most sample conditions, regardless of normalization with the combination of two (*RAD1a+RPS18*), three (*RAD1a+RPS18+ARF1*), and four (*RAD1a+RPS18+ARF1+RPS5*) reference genes (Fig 4). In the carbaryl-treated head, carbaryl-treated gut, and flupyradifurone-treated fat body, expression levels of *AChE1* normalized with two genes were not statistically similar with those computed with three reference gene combinations (Fig 4), but the lowest values of pairwise variation were V_2_/V_3_ in these condition (Fig 3), suggesting that selection of two genes is the most optimal in these conditions. Taken together, to compare target gene expression levels across different body parts of honey bees exposed to various pesticides, the combination of two genes (*RAD1a+RPS18*) is sufficient for target gene normalization.

A small number of reference genes might be suitable if target gene expression levels calculated by the combination of a different number of multiple reference genes are not significantly different, which also reduces the financial and technical burden in experiments; therefore, the application of a single reference gene has also been suggested in the previous studies after comparison of target gene expression normalized with multiple genes and a single gene [28, 30, 63]. Similarly, in this study, *AChE1* expression levels normalized with a single gene among *RPS5*, *RPS18*, and *GAPDH* were generally similar to those normalized with the combination of multiple reference genes (Fig 4). However, in the carbaryl-treated head, *RPS18* and *GAPDH* were not suggested as single reference genes because expression levels of *AChE1* normalized with one of these two genes were significantly different from those calculated with multiple reference genes (Fig 4). Furthermore, in the carbaryl-treated gut, no single gene demonstrated an expression level of *AChE1* that is similar to those calculated with multiple reference genes (Fig 4). Based on the comprehensive results analyzed by expression levels of *AChE1*, a single gene is not sufficient but the combination of two genes (*RAD1a+RPS18*) is more appropriately applied as the common reference genes for comparison of target gene expression levels across different honey bee samples.

In addition to RefFinder, three software also analyzed that *RAD1a* and *RPS18* were ranked as the most stable (Fig 2). Based on the criteria: mean stability < 0.15 in NormFinder [57, 58], SD value < 1.0 in BestKeeper [59], and M < 0.5 in geNorm [60, 61], the calculated values of both genes were below the cut-off values (Fig 2 and Tables 1 and S2–S4). C_q_ distribution analysis also revealed CV values < 1, which indicates low variance (Fig 1) [56], suggesting that the combination of *RAD1a* and *RPS18* can be selected as reference genes for target gene normalization in different body parts from honey bees exposed to various pesticides.

*RPS18* and *RAD1a* have been widely investigated as the optimal reference gene in honey bees under various conditions, such as different seasons (12 months), in different tissues (head, thorax, and abdomen), in adult labor roles (nurse and forager), in different developmental stages, pesticide treatments, and under bacterial challenge [27–30, 32, 68], further supporting the suggestion that *RAD1a* and *RPS18* are the most appropriate reference genes in honey bee studies.

Based on the analysis of reference gene expression stability with C_q_ distribution (Fig 1), taken together, four programs (Table 1 and Fig 2), pairwise variation (Fig 3), and target gene normalization (Fig 4), the combination of *RAD1a* and *RPS18* is suggested to be used as the most suitable method for normalization of target gene expression levels in the qRT-PCR assay in different body parts of honey bees exposed to various pesticides.

### Expression profiles of target genes in body parts of pesticide-exposed honey bees

As summarized in the integrated results of reference gene validation, since the combination of *RAD1a* and *RPS18* was determined to be the optimal reference gene set in this study (Figs 1–4 and Table 1), expression profiles of genes putatively associated with the pesticide detoxification process were investigated in the body parts of honey bees exposed to seven pesticides.

Pesticides are known to generate oxidative stress through ROS production in various animals, including humans and insects [69]. Antioxidant enzymes, such as *SOD*, *CAT*, glutathione S-transferase (*GST*), glutathione peroxidase (*GPx*), and glutathione reductase, play critical roles in defense against oxidative stress in organisms [70–74] and are also associated with pesticide detoxification in insects [75]. In particular, *SOD*, *CAT*, *GST*, and peroxidase have been identified as the most important antioxidant enzymes in honey bees [76–78]. Among these enzymes, the activity of the *GPx* and *CAT* was elevated by imidacloprid exposure in *A*. *mellifera* [79]. Furthermore, in *A*. *dorsata* and *A*. *cerana*, three different pesticides, dimethoate (OP), cypermethrin (pyrethroid), and endosulfan (organochlorine), significantly increased the enzymatic activity of *CAT* and *SOD* [80]. These studies indicate that oxidative stress might be induced by exposure to various pesticides in honey bees. In the present study, the transcription of *CAT* and *SOD1* was induced in the gut, fat body, and carcass, although their expression levels varied compared with the control group (Fig 5).

Interestingly, the expression of *SOD1* in the head and thorax was induced by exposure to seven and four pesticides, respectively, but the expression levels of *CAT* were significantly reduced by five pesticides (acetamiprid, imidacloprid, flupyradifurone, fenitrothion, and carbaryl) in the thorax (Fig 5). Moreover, in the head, *CAT* expression was significantly reduced by imidacloprid but was significantly increased by exposure to fenitrothion, carbaryl, amitraz, and bifenthrin (Fig 5). Similar large variations in antioxidant enzyme activities, including *SOD*, *CAT*, and *GST*, were investigated in different tissues (head, midgut, and abdomen) of honey bees exposed to imidacloprid, glyphosate, and difenoconazole, alone and in binary and ternary mixtures [81]. This suggests that antioxidant enzymes might respond differently in different body parts and to different types of pesticides. However, it seems clear that *CAT* and *SOD* were induced more in the gut, fat body, and carcass than in the head and thorax (Fig 5). Because the honey bees were fed sucrose solution containing pesticides in this study, the gut would be the primary organ directly exposed to pesticides. In addition, the midgut is one of the main sites of detoxification in insects [82]. A recent study revealed that the expression of key enzymes of the honey bee xenobiotic detoxification pathway is promoted by the gut microbiota [83]. After being metabolized in the midgut epithelial cells, pesticides are transported into the hemolymph across the basal membrane [84]; and the fat body plays an essential role in detoxification processes in insects [85]. In addition, although the detoxification pathway that occurs in the carcass, including the epidermis, has not been well characterized, the *CYP* gene involved in oxidative stress responses was also found to be abundantly expressed in the carcass or epidermis of *A*. *cerana* [86]. These studies support the findings of the present study that genes encoding antioxidant enzymes were more induced in the gut, fat body, and carcass than in the head and thorax (Fig 5).

Similar to *CAT* and *SOD1*, the induction ratios of *CYP9Q1*, *CYP9Q2*, and *CYP9Q3* varied depending on the body parts or pesticides; however, they generally exhibited higher expression in the gut, fat body, and carcass than in the head and thorax (Fig 5). *CYP* enzymes contribute to honey bee detoxification [87]. In particular, *CYP9Q1*, *CYP9Q2*, and *CYP9Q3* were recognized to efficiently metabolize fluvalinate, a typical pyrethroid pesticide that has been widely used as an acaricide for mite control in honey bee colonies [45]. In addition, transgenic *Drosophila* lines artificially expressing honey bee *CYP9Q3* exhibited significant resistance to thiacloprid compared to the control flies [44], suggesting that *CYP9Q* subfamily members contribute significantly to honey bee xenobiotic detoxification and pesticide tolerance. However, the expression of *CYP9Qs* did not show significant trends across pesticides or body parts of honey bees in this study (Fig 5). According to a previous study comparing transcript levels of *CYP9Q1*, *CYP9Q2*, and *CYP9Q3* in honey bees after three pyrethroid treatments, *CYP9Q2* transcripts were increased by the three pyrethroids. However, tau-fluvalinate and cypermethrin repressed the expression of *CYP9Q1*, whereas they enhanced the expression of *CYP9Q3*. In contrast, bifenthrin did not induce the expression of *CYP9Q3* but induced the transcription of *CYP9Q1* [45]. While insecticides belonging to the same family have different effects on the expression of the three *CYP9Qs* [45], the same expression pattern of *CYP9Qs* cannot be expected in honey bees exposed to insecticides belonging to different families.

According to the expression profiles of *AChE1* in the five body parts, *AChE1* also did not exhibit significant patterns for different body parts or pesticides (Fig 5). According to previous studies, honey bees possess two different *AChEs*, *AChE1* and *AChE2*; *AChE1* is soluble and shows little enzymatic activity in non-neuronal tissues, whereas *AChE2* is a membrane-anchored form with high catalytic activity in neuronal tissues [42, 88]. In the kinetic analysis of *in vitro* expressed *AChE1* and *AChE2*, *AChE1* reduced the inhibition of *AChE2* by OP and CB insecticides, suggesting a physiological function of *AChE1* as a bio-scavenger that provides chemical defense in honey bees [42]. The role of soluble *AChE* as a chemical defense has also been observed in nematodes [89] and *Drosophila* [90]. In particular, the expression of soluble *AChE* was statistically induced by dichlorvos treatment in *D*. *melanogaster* [90], further suggesting that soluble *AChE* exerts a chemical defense effect against xenobiotics. In contrast to these previous studies [42, 89, 90] showing the putative function of soluble *AChE* associated with defense against OP or CB pesticides, a more recent study revealed that imidacloprid and fluvalinate did not induce the expression of *AChE1* at the transcriptional or protein levels in the head and abdomen of honey bees [41]. In this study, *AChE1* was not induced by acetamiprid, fenitrothion, amitraz, and bifenthrin in all body parts. In contrast, imidacloprid induced *AChE1* expression levels in the head and gut, while carbaryl affected expression levels of *AChE1* in the head and carcass. In the fat body, the level of *AChE1* was higher than control after flupyradifurone exposure (Fig 5). This result indicates that the expression variability of *AChE1* depends on different body parts and pesticide treatments.

In conclusion, the expression of genes putatively involved in the detoxification mechanism did not exhibit significantly different expression patterns across different body parts or pesticides in honey bees. Given the high probability that honey bees are exposed to pesticides by their feeding activities [91, 92], gut and fat bodies were suggested to be optimal body parts to investigate the damage to honey bees by pesticide exposure based on determining the expression of genes, as molecular markers associated with detoxification metabolism. In particular, *SOD1* in the head and the fat body was significantly induced by seven pesticides, suggesting that *SOD1* could be a candidate molecular marker. However, *SOD1* is a typical antioxidant enzyme, and oxidative stress is generated by various stressors, such as chemicals [79–81], heavy metals [50], flight, and age [93] in honey bees; therefore, caution is needed when using *SOD1* as a molecular marker to investigate the damage caused by pesticides.

## Supporting information

S1 Fig Amplification specificities of primers used in this study. Agarose gel showing a single band with the expected size of PCR products amplified with designed primers (A). Melt curves analyzed by qRT-PCR for PCR products amplified with designed primers.
(TIF)

S2 Fig Comparison of target gene expression in honey bee head exposed to pesticides. Expression levels of *AChE1* (A), *CYP9Q1* (B), *CYP9Q2* (C), *CYP9Q3* (D), *CAT* (E), and *SOD1* (F) were normalized with the combination of *RAD1a* and *RPS18* in the head body part from honey bees treated with seven different pesticides. The difference of expression between the control and pesticide-treated sample were statistically compared with the independent samples T-test with Tukey’s comparison analysis, and significant differences were measured with *(*p* < 0.05), **(*p* < 0.01), and ***(*p* < 0.001).
(TIF)

S3 Fig Comparison of target gene expression in honey bee thorax exposed to pesticides. Expression levels of *AChE1* (A), *CYP9Q1* (B), *CYP9Q2* (C), *CYP9Q3* (D), *CAT* (E), and *SOD1* (F) were normalized with the combination of *RAD1a* and *RPS18* in the thorax body part from honey bees treated with seven different pesticides. The difference of expression between the control and pesticide-treated sample were statistically compared with the independent samples T-test with Tukey’s comparison analysis, and significant differences were measured with *(*p* < 0.05), **(*p* < 0.01), and ***(*p* < 0.001).
(TIF)

S4 Fig Comparison of target gene expression in honey bee gut exposed to pesticides. Expression levels of *AChE1* (A), *CYP9Q1* (B), *CYP9Q2* (C), *CYP9Q3* (D), *CAT* (E), and *SOD1* (F) were normalized with the combination of *RAD1a* and *RPS18* in the gut body part of honey bees treated with seven different pesticides. The difference of expression between the control and pesticide-treated sample were statistically compared with the independent samples T-test with Tukey’s comparison analysis, and significant differences were measured with *(*p* < 0.05), **(*p* < 0.01), and ***(*p* < 0.001).
(TIF)

S5 Fig Comparison of target gene expression in honey bee fat body exposed to pesticides. Expression levels of *AChE1* (A), *CYP9Q1* (B), *CYP9Q2* (C), *CYP9Q3* (D), *CAT* (E), and *SOD1* (F) were normalized with the combination of *RAD1a* and *RPS18* in the fat body part of honey bees treated with seven different pesticides. The difference of expression between the control and pesticide-treated sample were statistically compared with the independent samples T-test with Tukey’s comparison analysis, and significant differences were measured with *(*p* < 0.05), **(*p* < 0.01), and ***(*p* < 0.001).
(TIF)

S6 Fig Comparison of target gene expression in honey bee carcass exposed to pesticides. Expression levels of *AChE1* (A), *CYP9Q1* (B), *CYP9Q2* (C), *CYP9Q3* (D), *CAT* (E), and *SOD1* (F) were normalized with the combination of *RAD1a* and *RPS18* in the carcass body part of honey bees treated with seven different pesticides. The difference in expression between the control and pesticide-treated sample were statistically compared with the independent samples T-test with Tukey’s comparison analysis, and significant differences were measured with *(*p* < 0.05), **(*p* < 0.01), and ***(*p* < 0.001).
(TIF)

S1 Table Information of the primers and amplicons of the reference genes and target genes for qRT-PCR assay. (DOCX)

S2 Table Ranking and stability values of reference genes were calculated using NormFinder in different body parts treated with seven pesticides. (DOCX)

S3 Table Ranking and standard deviation of reference genes calculated using BestKeeper in different body parts treated with seven pesticides. (DOCX)

S4 Table Ranking and average expression stability M of reference genes calculated using geNorm in different body parts treated with seven pesticides. (DOCX)

S5 Table Ranking and geomean of the ranking value of reference genes calculated using RefFinder in different body parts treated with seven pesticides. (DOCX)
