# Supplementary material for: Validation of quantitative real-time PCR reference genes and spatial expression profiles of detoxication-related genes under pesticide induction in honey bee, Apis mellifera
Source: PLoS One. 2022 Nov 10;17(11):e0277455. doi: 10.1371/journal.pone.0277455 (PMC9648776; doi:10.1371/journal.pone.0277455)
Supplement: S1 Table — (DOCX) [file pone.0277455.s007.docx]

**Table S1**. Information of primers and amplicons of the reference genes and target genes for qRT-PCR assay

| **Gene** | | |  | **Primers** | | | | |  | **Amplicons** | | | |  | **Reference** |
| --- | --- | --- | --- | --- | --- | --- | --- | --- | --- | --- | --- | --- | --- | --- | --- |
| **Symbol** | **Full gene name** | **Accession no.** |  | **Sequence (5'->3')** | | **Size**  **(bp)** | **GC**  **(%)** | **Tm**  **(℃)** |  | **Size**  **(bp)*** | **GC**  **(%)** | **Efficiency**  **(%)** | **R²** |  |  |
| *RPS5* | 40S ribosomal protein S5 | XM_006570237 |  | Forward | GATGTTTCTCCGTTACGACGAGT | 23 | 48 | 62.9 |  | 114 | 45 | 92 | 0.999 |  | Jeon, et al. 2020 |
|  |  |  |  | Reverse | GAGTTCATCGGCTAAACATTCGG | 23 | 48 | 62.9 |  |  |  |  |  |  |  |
| *RPS18* | 40S ribosomal protein S18 | XM_625101 |  | Forward | GATTCCCGATTGGTTTTTGAATAG | 24 | 38 | 60.3 |  | 152 (446) | 35.5 | 107.6 | 0.999 |  | Moon et al. 2018 |
|  |  |  |  | Reverse | AACCCCAATAATGACGCAAACC | 22 | 45 | 60.1 |  |  |  |  |  |  |  |
| *GAPDH* | Glyceraldehyde-3-phosphate dehydrogenase | XM_393605 |  | Forward | CACCTTCTGCAAAATTATGGCG | 22 | 45 | 60.1 |  | 188 | 43.1 | 95.5 | 0.997 |  |  |
|  |  |  |  | Reverse | ACCTTTGCCAAGTCTAACTGTTAA | 24 | 38 | 60.3 |  |  |  |  |  |  |  |
| *ARF1* | ADP-ribosylation factor 1 | LOC409481 |  | Forward | GGGCTTCATTCTCTCCGCAA | 20 | 55 | 60.5 |  | 91 | 47.3 | 98.1 | 0.994 |  | Kim et al.2021 |
|  |  |  |  | Reverse | AGAGCCAATCAAGACCCTCG | 20 | 55 | 60.5 |  |  |  |  |  |  |  |
| *RAD1A* | Ras-related protein Rab-1A | LOC102654987 |  | Forward | CTTAGAGTGGGTCCTCCATC | 20 | 55 | 60.5 |  | 101 | 42.6 | 97.33 | 0.996 |  |  |
|  |  |  |  | Reverse | CAGCAGCATCCAGATTTAGAGG | 22 | 50 | 62.1 |  |  |  |  |  |  |  |
| *AChE1* | Acetylcholinesterase1 | XM_016914793 |  | Forward | GAAAGAGACGTGCAGCGGTA | 20 | 55 | 60.5 |  | 136 | 59.6 | 95 | 0.999 |  | In this study |
|  |  |  |  | Reverse | ATCGAATATCTCCGGTACCTCG | 22 | 50 | 62.1 |  |  |  |  |  |  |  |
| *CAT* | Catalase | NM_001178069 |  | Forward | CTTGGCCCAAACAATCTGCAAT | 22 | 45 | 60.3 |  | 151 | 37.7 | 98.43 | 0.999 |  | Gizaw et al. 2020 |
|  |  |  |  | Reverse | GACATTCTCTAGGCCCACCA | 20 | 55 | 60.5 |  |  |  |  |  |  |  |
| *CYP9Q1* | Cytochrome P450 9Q1 | XM_006562301 |  | Forward | ACCTGTCCACGAGGAATCAC | 20 | 55 | 60.5 |  | 234 | 57.3 | 96.73 | 0.999 |  |  |
|  |  |  |  | Reverse | CCTTCACCCCGATCGTCTTT | 20 | 55 | 60.5 |  |  |  |  |  |  |  |
| *CYP9Q2* | Cytochrome P450 9Q2 | XM_392000 |  | Forward | ACCTGATCAAGACCATCACGAT | 22 | 45 | 60.1 |  | 170 | 54.1 | 92.47 | 0.999 |  |  |
|  |  |  |  | Reverse | GATCTTGCTCGAGGTGAAGG | 20 | 55 | 60.5 |  |  |  |  |  |  |  |
| *CYP9Q3* | Cytochrome P450 9Q3 | XM_006562300 |  | Forward | TACGTGGGCATTTACGAGTTCA | 22 | 45 | 60.1 |  | 249 | 53.4 | 95.12 | 0.999 |  |  |
|  |  |  |  | Reverse | CTCGGTCATCAGCTTGAACATA | 22 | 45 | 60.1 |  |  |  |  |  |  |  |
| *SOD1* | Superoxide dismutase 1 | NM_001178027 |  | Forward | GCGTTCTTCAGGGTGAAGTC | 20 | 55 | 60.5 |  | 203 | 41.9 | 91.77 | 0.996 |  |  |
|  |  |  |  | Reverse | ATCAGGTCCACCATGATCCTTT | 22 | 45 | 60.5 |  |  |  |  |  |  |  |

* Numbers in brackets indicate the size of the PCR product amplified with genomic DNA.
