# Supplementary material for: Validation of quantitative real-time PCR reference genes and spatial expression profiles of detoxication-related genes under pesticide induction in honey bee, Apis mellifera
Source: PLoS One. 2022 Nov 10;17(11):e0277455. doi: 10.1371/journal.pone.0277455 (PMC9648776; doi:10.1371/journal.pone.0277455)
Supplement: S2 Table — (DOCX) [file pone.0277455.s008.docx]

**Table S2**. Ranking and stability values of reference genes calculated using NormFinder in different body parts treated with seven pesticides.

| **Body**  **part** | **Rank** | **Pesticide** | | | | | | | | |
| --- | --- | --- | --- | --- | --- | --- | --- | --- | --- | --- |
|  |  | **All** | **Control** | **Acetamiprid** | **Imidacloprid** | **Flupyradifurone** | **Fenitrothion** | **Carbaryl** | **Amitraz** | **Bifenthrin** |
| **Head** | 1 | *RPS5* (0.003) | *GAPDH* (0.004) | *RPS5* (0.000) | *RPS5* (0.002) | *RPS18* (0.001) | *GAPDH* (0.002) | *GAPDH* (0.001) | *ARF1* (0.001) | *RAD1a* (0.001) |
|  | 2 | *RPS18* (0.003) | *RPS5* (0.008) | *RAD1a* (0.000) | *RPS18* (0.006) | *RPS5* (0.002) | *ARF1* (0.004) | *RPS5* (0.002) | *RPS18* (0.002) | *RPS5* (0.002) |
|  | 3 | *RAD1a* (0.009) | *ARF1* (0.011) | *GAPDH* (0.003) | *RAD1a* (0.007) | *ARF1* (0.004) | *RPS18* (0.006) | *ARF1* (0.002) | *RAD1a* (0.004) | *ARF1* (0.007) |
|  | 4 | *ARF1* (0.021) | *RAD1a* (0.013) | *RPS18* (0.004) | *GAPDH* (0.011) | *RAD1a* (0.004) | *RPS5* (0.010) | *RAD1a* (0.004) | *RPS5* (0.008) | *GAPDH* (0.009) |
|  | 5 | *GAPDH* (0.028) | *RPS18* (0.014) | *ARF1* (0.005) | *ARF1* (0.012) | *GAPDH* (0.005) | *RAD1a* (0.011) | *RPS18* (0.008) | *GAPDH* (0.014) | *RPS18* (0.011) |
| **Thorax** | 1 | *RPS5* (0.013) | *RAD1a* (0.003) | *RAD1a* (0.001) | *RPS5* (0.003) | *ARF1* (0.003) | *RPS5* (0.024) | *ARF1* (0.001) | *RPS5* (0.007) | *RPS5* (0.006) |
|  | 2 | *RPS18* (0.013) | *RPS18* (0.004) | *ARF1* (0.003) | *RAD1a* (0.005) | *RPS18* (0.004) | *RPS18* (0.030) | *RPS18* (0.002) | *RPS18* (0.016) | *RPS18* (0.006) |
|  | 3 | *ARF1* (0.041) | *RPS5* (0.004) | *RPS18* (0.007) | *RPS18* (0.008) | *RAD1a* (0.006) | *ARF1* (0.066) | *RAD1a* (0.004) | *RAD1a* (0.027) | *RAD1a* (0.027) |
|  | 4 | *RAD1a* (0.056) | *ARF1* (0.011) | *RPS5* (0.026) | *ARF1* (0.018) | *RPS5* (0.021) | *RAD1a* (0.165) | *RPS5* (0.007) | *ARF1* (0.086) | *ARF1* (0.055) |
|  | 5 | *GAPDH* (0.166)* | *GAPDH* (0.021) | *GAPDH* (0.057) | *GAPDH* (0.032) | *GAPDH* (0.049) | *GAPDH** (0.309)* | *GAPDH* (0.025) | *GAPDH** (0.262)* | *GAPDH** (0.156)* |
| **Gut** | 1 | *RPS5* (0.005) | *GAPDH* (0.003) | *RPS18* (0.002) | *RAD1a* (0.002) | *RPS5* (0.001) | *RPS5* (0.001) | *ARF1* (0.001) | *ARF1* (0.001) | *RPS18* (0.002) |
|  | 2 | *RAD1a* (0.006) | *RAD1a* (0.003) | *GAPDH* (0.005) | *RPS5* (0.006) | *ARF1* (0.001) | *RPS18* (0.001) | *RAD1a* (0.002) | *RAD1a* (0.001) | *GAPDH* (0.010) |
|  | 3 | *ARF1* (0.010) | *RPS5* (0.013) | *RAD1a* (0.005) | *RPS18* (0.008) | *RAD1a* (0.004) | *RAD1a* (0.002) | *RPS5* (0.004) | *RPS18* (0.004) | *RPS5* (0.010) |
|  | 4 | *RPS18* (0.011) | *RPS18* (0.018) | *RPS5* (0.006) | *ARF1* (0.008) | *RPS18* (0.006) | *ARF1* (0.003) | *GAPDH* (0.005) | *GAPDH* (0.006) | *ARF1* (0.012) |
|  | 5 | *GAPDH* (0.018) | *ARF1* (0.023) | *ARF1* (0.010) | *GAPDH* (0.015) | *GAPDH* (0.015) | *GAPDH* (0.005) | *RPS18* (0.011) | *RPS5* (0.008) | *RAD1a* (0.020) |
| **Fat body** | 1 | *RAD1a* (0.005) | *RAD1a* (0.008) | *RPS18* (0.001) | *RPS5* (0.002) | *RPS18* (0.000) | *RPS18* (0.002) | *RPS18* (0.001) | *RPS5* (0.001) | *RPS18* (0.001) |
|  | 2 | *ARF1* (0.007) | *ARF1* (0.015) | *GAPDH* (0.001) | *ARF1* (0.002) | *ARF1* (0.001) | *ARF1* (0.005) | *RAD1a* (0.001) | *ARF1* (0.001) | *ARF1* (0.001) |
|  | 3 | *RPS5* (0.008) | *RPS5* (0.021) | *RAD1a* (0.003) | *RPS18* (0.007) | *GAPDH* (0.005) | *GAPDH* (0.007) | *RPS5* (0.003) | *RAD1a* (0.001) | *RPS5* (0.004) |
|  | 4 | *RPS18* (0.013) | *RPS18* (0.027) | *RPS5* (0.004) | *RAD1a* (0.008) | *RAD1a* (0.006) | *RAD1a* (0.008) | *ARF1* (0.006) | *RPS18* (0.007) | *GAPDH* (0.004) |
|  | 5 | *GAPDH* (0.018) | *GAPDH* (0.034) | *ARF1* (0.007) | *GAPDH* (0.016) | *RPS5* (0.008) | *RPS5* (0.011) | *GAPDH* (0.008) | *GAPDH* (0.016) | *RAD1a* (0.015) |
| **Carcass** | 1 | *RAD1a* (0.005) | *RPS18* (0.002) | *ARF1* (0.001) | *ARF1* (0.001) | *GAPDH* (0.002) | *GAPDH* (0.004) | *RAD1a* (0.001) | *ARF1* (0.001) | *ARF1* (0.003) |
|  | 2 | *RPS5* (0.005) | *RPS5* (0.004) | *RAD1a* (0.001) | *RPS5* (0.001) | *RAD1a* (0.005) | *RAD1a* (0.005) | *ARF1* (0.002) | *RPS5* (0.003) | *RAD1a* (0.003) |
|  | 3 | *RPS18* (0.007) | *ARF1* (0.005) | *RPS18* (0.005) | *RPS18* (0.003) | *RPS5* (0.008) | *RPS18* (0.006) | *RPS5* (0.002) | *GAPDH* (0.004) | *RPS5* (0.004) |
|  | 4 | *GAPDH* (0.011) | *RAD1a* (0.005) | *RPS5* (0.006) | *RAD1a* (0.004) | *RPS18* (0.013) | *RPS5* (0.012) | *RPS18* (0.004) | *RAD1a* (0.004) | *GAPDH* (0.006) |
|  | 5 | *ARF1* (0.011) | *GAPDH* (0.012) | *GAPDH* (0.015) | *GAPDH* (0.005) | *ARF1* (0.015) | *ARF1* (0.017) | *GAPDH* (0.009) | *RPS18* (0.007) | *RPS18* (0.010) |

* Stability values exceeding the cut-off value (0.15).
