# Supplementary material for: Validation of quantitative real-time PCR reference genes and spatial expression profiles of detoxication-related genes under pesticide induction in honey bee, Apis mellifera
Source: PLoS One. 2022 Nov 10;17(11):e0277455. doi: 10.1371/journal.pone.0277455 (PMC9648776; doi:10.1371/journal.pone.0277455)
Supplement: S3 Table — (DOCX) [file pone.0277455.s009.docx]

**Table S3**. Ranking and standard deviation of reference genes calculated using BestKeeper in different body parts treated with seven pesticides.

| **Body part** | **Rank** | **Pesticide** | | | | | | | | |
| --- | --- | --- | --- | --- | --- | --- | --- | --- | --- | --- |
|  |  | **All** | **Control** | **Acetamiprid** | **Imidacloprid** | **Flupyradifurone** | **Fenitrothion** | **Carbaryl** | **Amitraz** | **Bifenthrin** |
| **Head** | 1 | *ARF1* (0.291) | *RPS5* (0.133) | *ARF1* (0.138) | *ARF1* (0.178) | *ARF1* (0.027) | *GAPDH* (0.058) | *ARF1* (0.089) | *RPS5* (0.044) | *RPS5* (0.060) |
|  | 2 | *RAD1a* (0.407) | *RPS18* (0.180) | *RAD1a* (0.153) | *RAD1a* (0.187) | *RPS5* (0.051) | *ARF1* (0.096) | *RPS5* (0.104) | *RAD1a* (0.087) | *GAPDH* (0.064) |
|  | 3 | *RPS18* (0.512) | *GAPDH* (0.189) | *RPS5* (0.162) | *RPS5* (0.218) | *RAD1a* (0.053) | *RPS18* (0.100) | *RAD1a* (0.107) | *RPS18* (0.127) | *RAD1a* (0.076) |
|  | 4 | *RPS5* (0.536) | *ARF1* (0.222) | *GAPDH* (0.187) | *RPS18* (0.284) | *RPS18* (0.053) | *RAD1a* (0.122) | *GAPDH* (0.109) | *ARF1* (0.140) | *ARF1* (0.144) |
|  | 5 | *GAPDH* (0.765) | *RAD1a* (0.273) | *RPS18* (0.218) | *GAPDH* (0.327) | *GAPDH* (0.069) | *RPS5* (0.147) | *RPS18* (0.167) | *GAPDH* (0.256) | *RPS18* (0.156) |
| **Thorax** | 1 | *ARF1* (0.706) | *ARF1* (0.100) | *RPS5* (0.162) | *ARF1* (0.484) | *RPS5* (0.436) | *RAD1a* (0.330) | *RPS5* (0.487) | *ARF1* (0.696) | *RAD1a* (1.231)* |
|  | 2 | *RAD1a* (0.724) | *RAD1a* (0.149) | *ARF1* (0.318) | *RAD1a* (0.571) | *RAD1a* (0.558) | *ARF1* (1.073)* | *RAD1a* (0.542) | *RAD1a* (1.151)* | *ARF1* (1.242)* |
|  | 3 | *RPS5* (0.834) | *RPS5* (0.211) | *RAD1a* (0.318) | *RPS18* (0.587) | *ARF1* (0.671) | *RPS5* (1.769)* | *RPS18* (0.571) | *RPS5* (1.293)* | *RPS5* (1.371)* |
|  | 4 | *RPS18* (1.037)* | *RPS18* (0.240) | *RPS18* (0.504) | *RPS5* (0.658) | *RPS18* (0.691) | *RPS18* (2.827)* | *ARF1* (0.587) | *RPS18* (1.740)* | *RPS18* (1.516)* |
|  | 5 | *GAPDH* (2.865)* | *GAPDH* (0.391) | *GAPDH* (0.809) | *GAPDH* (0.822) | *GAPDH* (0.956) | *GAPDH* (6.267)* | *GAPDH* (0.693) | *GAPDH* (5.364)* | *GAPDH* (3.753)* |
| **Gut** | 1 | *RPS18* (0.433) | *RAD1a* (0.076) | *RAD1a* (0.242) | *RPS5* (0.384) | *RPS18* (0.160) | *RPS18* (0.231) | *GAPDH* (0.038) | *GAPDH* (0.080) | *ARF1* (0.416) |
|  | 2 | *RPS5* (0.474) | *GAPDH* (0.084) | *ARF1* (0.249) | *ARF1* (0.404) | *RAD1a* (0.198) | *RAD1a* (0.236) | *ARF1* (0.047) | *RPS5* (0.122) | *RPS5* (0.427) |
|  | 3 | *RAD1a* (0.515) | *RPS5* (0.133) | *RPS18* (0.307) | *RPS18* (0.404) | *RPS5* (0.218) | *ARF1* (0.253) | *RAD1a* (0.047) | *RAD1a* (0.133) | *RPS18* (0.440) |
|  | 4 | *ARF1* (0.533) | *RPS18* (0.204) | *GAPDH* (0.331) | *RAD1a* (0.491) | *ARF1* (0.271) | *RPS5* (0.260) | *RPS5* (0.049) | *ARF1* (0.142) | *GAPDH* (0.600) |
|  | 5 | *GAPDH* (0.701) | *ARF1* (0.316) | *RPS5* (0.367) | *GAPDH* (0.629) | *GAPDH* (0.418) | *GAPDH* (0.284) | *RPS18* (0.156) | *RPS18* (0.162) | *RAD1a* (0.624) |
| **Fat body** | 1 | *RPS18* (0.205) | *RPS18* (0.053) | *ARF1* (0.044) | *RPS18* (0.096) | *RPS5* (0.338) | *RPS5* (0.153) | *RPS5* (0.009) | *RPS18* (0.180) | *RPS5* (0.091) |
|  | 2 | *RPS5* (0.284) | *RPS5* (0.120) | *RPS18* (0.060) | *RPS5* (0.169) | *RPS18* (0.373) | *RPS18* (0.213) | *RPS18* (0.049) | *RPS5* (0.213) | *GAPDH* (0.111) |
|  | 3 | *ARF1* (0.353) | *RAD1a* (0.422) | *GAPDH* (0.064) | *RAD1a* (0.191) | *GAPDH* (0.453) | *ARF1* (0.229) | *RAD1a* (0.057) | *ARF1* (0.258) | *RPS18* (0.127) |
|  | 4 | *RAD1a* (0.373) | *ARF1* (0.724) | *RAD1a* (0.080) | *ARF1* (0.227) | *ARF1* (0.456) | *GAPDH* (0.298) | *ARF1* (0.098) | *RAD1a* (0.264) | *ARF1* (0.169) |
|  | 5 | *GAPDH* (0.447) | *GAPDH* (0.824) | *RPS5* (0.096) | *GAPDH* (0.338) | *RAD1a* (0.522) | *RAD1a* (0.327) | *GAPDH* (0.102) | *GAPDH* (0.424) | *RAD1a* (0.320) |
| **Carcass** | 1 | *ARF1* (0.379) | *GAPDH* (0.333) | *ARF1* (0.036) | *RPS18* (0.076) | *ARF1* (0.060) | *ARF1* (0.276) | *RPS18* (0.082) | *RPS18* (0.044) | *GAPDH* (0.571) |
|  | 2 | *RAD1a* (0.443) | *ARF1* (0.333) | *RAD1a* (0.036) | *GAPDH* (0.084) | *RAD1a* (0.191) | *RAD1a* (0.327) | *RPS5* (0.098) | *ARF1* (0.062) | *RAD1a* (0.687) |
|  | 3 | *RPS18* (0.453) | *RPS18* (0.369) | *RPS18* (0.098) | *ARF1* (0.098) | *GAPDH* (0.209) | *GAPDH* (0.336) | *RAD1a* (0.136) | *GAPDH* (0.064) | *RPS5* (0.711) |
|  | 4 | *RPS5* (0.455) | *RPS5* (0.371) | *RPS5* (0.109) | *RPS5* (0.102) | *RPS5* (0.340) | *RPS18* (0.389) | *ARF1* (0.153) | *RPS5* (0.073) | *ARF1* (0.716) |
|  | 5 | *GAPDH* (0.490) | *RAD1a* (0.456) | *GAPDH* (0.138) | *RAD1a* (0.147) | *RPS18* (0.391) | *RPS5* (0.456) | *GAPDH* (0.233) | *RAD1a* (0.087) | *RPS18* (0.756) |

* Standard deviations of genes exceed the cut-off value (1.0).
