# Supplementary material for: Validation of quantitative real-time PCR reference genes and spatial expression profiles of detoxication-related genes under pesticide induction in honey bee, Apis mellifera
Source: PLoS One. 2022 Nov 10;17(11):e0277455. doi: 10.1371/journal.pone.0277455 (PMC9648776; doi:10.1371/journal.pone.0277455)
Supplement: S4 Table — (DOCX) [file pone.0277455.s010.docx]

**Table S4**. Ranking and average expression stability M of reference genes calculated using geNorm in different body parts treated with seven pesticides.

| **Body part** | **Rank** | **Pesticide** | | | | | | | | |
| --- | --- | --- | --- | --- | --- | --- | --- | --- | --- | --- |
|  |  | **All** | **Control** | **Acetamiprid** | **Imidacloprid** | **Flupyradifurone** | **Fenitrothion** | **Carbaryl** | **Amitraz** | **Bifenthrin** |
| **Head** | 1 | *RPS5* (0.206) | *RAD1a* (0.183) | *RPS5* (0.033) | *RPS18* (0.091) | *RPS5* (0.049) | *RPS18* (0.090) | *GAPDH* (0.031) | *ARF1* (0.088) | *RAD1a* (0.105) |
|  | 2 | *RPS18* (0.213) | *ARF1* (0.185) | *RAD1a* (0.035) | *RPS5* (0.106) | *RAD1a* (0.058) | *RPS5* (0.103) | *ARF1* (0.034) | *RPS18* (0.090) | *RPS5* (0.116) |
|  | 3 | *RAD1a* (0.238) | *GAPDH* (0.208) | *GAPDH* (0.043) | *GAPDH* (0.111) | *RPS18* (0.065) | *GAPDH* (0.114) | *RAD1a* (0.036) | *RAD1a* (0.099) | *GAPDH* (0.122) |
|  | 4 | *ARF1* (0.298) | *RPS5* (0.258) | *RPS18* (0.065) | *RAD1a* (0.182) | *ARF1* (0.085) | *ARF1* (0.150) | *RPS5* (0.059) | *RPS5* (0.115) | *ARF1* (0.151) |
|  | 5 | *GAPDH* (0.410) | *RPS18* (0.280) | *ARF1* (0.085) | *ARF1* (0.217) | *GAPDH* (0.096) | *RAD1a* (0.185) | *RPS18* (0.105) | *GAPDH* (0.172) | *RPS18* (0.185) |
| **Thorax** | 1 | *RPS5* (0.583)* | *RAD1a* (0.105) | *ARF1* (0.131) | *RPS5* (0.168) | *ARF1* (0.142) | *RPS5* (0.347) | *RPS18* (0.039) | *ARF1* (0.214) | *RPS5* (0.402) |
|  | 2 | *RPS18* (0.589)* | *ARF1* (0.114) | *RAD1a* (0.132) | *RPS18* (0.184) | *RPS18* (0.164) | *ARF1* (0.424) | *RAD1a* (0.047) | *RPS5* (0.218) | *RPS18* (0.443) |
|  | 3 | *ARF1* (0.675)* | *RPS5* (0.118) | *RPS18* (0.169) | *RAD1a* (0.201) | *RAD1a* (0.183) | *RPS18* (0.456) | *ARF1* (0.050) | *RPS18* (0.277) | *RAD1a* (0.533)* |
|  | 4 | *RAD1a* (0.790)* | *RPS18* (0.144) | *RPS5* (0.259) | *ARF1* (0.235) | *RPS5* (0.253) | *RAD1a* (0.876)* | *RPS5* (0.078) | *RAD1a* (0.342) | *ARF1* (0.658)* |
|  | 5 | *GAPDH* (1.179)* | *GAPDH* (0.246) | *GAPDH* (0.465) | *GAPDH* (0.283) | *GAPDH* (0.401) | *GAPDH* (1.424)* | *GAPDH* (0.132) | *GAPDH* (0.683)* | *GAPDH* (1.235)* |
| **Gut** | 1 | *RPS5* (0.185) | *RAD1a* (0.190) | *GAPDH* (0.075) | *RPS5* (0.109) | *RAD1a* (0.058) | *RPS18* (0.023) | *RAD1a* (0.046) | *RPS18* (0.041) | *ARF1* (0.046) |
|  | 2 | *RPS18* (0.196) | *GAPDH* (0.194) | *RPS5* (0.075) | *RPS18* (0.120) | *RPS18* (0.063) | *RAD1a* (0.024) | *ARF1* (0.049) | *ARF1* (0.042) | *RPS5* (0.053) |
|  | 3 | *RAD1a* (0.211) | *RPS5* (0.226) | *RPS18* (0.077) | *ARF1* (0.143) | *RPS5* (0.066) | *ARF1* (0.028) | *GAPDH* (0.056) | *RAD1a* (0.044) | *RPS18* (0.058) |
|  | 4 | *ARF1* (0.237) | *RPS18* (0.252) | *RAD1a* (0.101) | *RAD1a* (0.167) | *ARF1* (0.095) | *RPS5* (0.035) | *RPS5* (0.086) | *GAPDH* (0.079) | *GAPDH* (0.174) |
|  | 5 | *GAPDH* (0.282) | *ARF1* (0.351) | *ARF1* (0.137) | *GAPDH* (0.217) | *GAPDH* (0.165) | *GAPDH* (0.052) | *RPS18* (0.134) | *RPS5* (0.110) | *RAD1a* (0.278) |
| **Fat body** | 1 | *RPS5* (0.213) | *RPS5* (0.061) | *GAPDH* (0.031) | *RPS5* (0.095) | *ARF1* (0.063) | *GAPDH* (0.090) | *RAD1a* (0.050) | *RAD1a* (0.057) | *RPS18* (0.048) |
|  | 2 | *RPS18* (0.213) | *RPS18* (0.069) | *RPS18* (0.035) | *RAD1a* (0.111) | *GAPDH* (0.068) | *RAD1a* (0.103) | *RPS18* (0.055) | *ARF1* (0.061) | *RPS5* (0.048) |
|  | 3 | *RAD1a* (0.222 | *RAD1a* (0.080) | *RPS5* (0.039) | *ARF1* (0.116) | *RAD1a* (0.079) | *RPS18* (0.118) | *ARF1* (0.065) | *RPS5* (0.066) | *GAPDH* (0.049) |
|  | 4 | *ARF1* (0.425) | *ARF1* (0.148) | *RAD1a* (0.062) | *RPS18* (0.134) | *RPS18* (0.113) | *ARF1* (0.149) | *RPS5* (0.084) | *RPS18* (0.095) | *ARF1* (0.065) |
|  | 5 | *GAPDH* (0.480) | *GAPDH* (0.173) | *ARF1* (0.094) | *GAPDH* (0.198) | *RPS5* (0.140) | *RPS5* (0.175) | *GAPDH* (0.113) | *GAPDH* (0.162) | *RAD1a* (0.163) |
| **Carcass** | 1 | *RPS5* (0.153) | *RPS18* (0.038) | *ARF1* (0.063) | *RPS5* (0.052) | *RPS5* (0.138) | *RPS18* (0.092) | *RPS5* (0.048) | *RPS5* (0.045) | *RPS5* (0.051) |
|  | 2 | *RPS18* (0.155) | *RPS5* (0.042) | *RAD1a* (0.063) | *RPS18* (0.061) | *RPS18* (0.151) | *RPS5* (0.114) | *RPS18* (0.057) | *GAPDH* (0.049) | *ARF1* (0.056) |
|  | 3 | *RAD1a* (0.164) | *ARF1* (0.047) | *RPS5* (0.075) | *RAD1a* (0.066) | *GAPDH* (0.171) | *GAPDH* (0.122) | *RAD1a* (0.062) | *ARF1* (0.058) | *RAD1a* (0.068) |
|  | 4 | *ARF1* (0.188) | *RAD1a* (0.089) | *RPS18* (0.083) | *ARF1* (0.075) | *RAD1a* (0.194) | *RAD1a* (0.170) | *ARF1* (0.082) | *RAD1a* (0.077) | *RPS18* (0.098) |
|  | 5 | *GAPDH* (0.211) | *GAPDH* (0.150) | *GAPDH* (0.161) | *GAPDH* (0.091) | *ARF1* (0.244) | *ARF1* (0.227) | *GAPDH* (0.112) | *RPS18* (0.107) | *GAPDH* (0.138) |

* Average expression stability M of the gene exceeds the cut-off value (0.5).
