# Supplementary material for: Validation of quantitative real-time PCR reference genes and spatial expression profiles of detoxication-related genes under pesticide induction in honey bee, Apis mellifera
Source: PLoS One. 2022 Nov 10;17(11):e0277455. doi: 10.1371/journal.pone.0277455 (PMC9648776; doi:10.1371/journal.pone.0277455)
Supplement: S5 Table — (DOCX) [file pone.0277455.s011.docx]

**Table S5**. Ranking and geomean of ranking value of reference genes calculated using RefFinder in different body parts treated with seven pesticides.

| **Body part** | **Rank** | **Pesticide** | | | | | | | | |
| --- | --- | --- | --- | --- | --- | --- | --- | --- | --- | --- |
|  |  | **All** | **Control** | **Acetamiprid** | **Imidacloprid** | **Flupyradifurone** | **Fenitrothion** | **Carbaryl** | **Amitraz** | **Bifenthrin** |
| **Head** | 1 | *RPS5* (1.414) | GAPDH (1.732) | *RAD1a* (1.414) | *RPS5* (1.316) | *RPS5* (1.414) | *GAPDH* (1.316) | *GAPDH* (1.414) | *ARF1* (1.414) | *RPS5* (1.189) |
|  | 2 | *RPS18* (1.861) | *RPS5* (2.000) | *RPS5* (1.565) | *RPS18* (2.000) | *RPS18* (2.060) | *RPS18* (2.060) | *ARF1* (1.565) | *RPS18* (1.861) | *RAD1a* (1.565) |
|  | 3 | *RAD1a* (2.711) | *ARF1* (2.449) | *GAPDH* (3.224) | *RAD1a* (2.913) | *RAD1a* (2.449) | *ARF1* (2.632) | *RPS5* (2.632) | *RAD1a* (2.711) | *GAPDH* (3.130) |
|  | 4 | *ARF1* (2.828) | *RAD1a* (2.991) | *ARF1* (3.344) | *ARF1* (3.344) | *ARF1* (2.828) | *RPS5* (2.991) | *RAD1a* (3.464) | *RPS5* (2.828) | *ARF1* (3.464) |
|  | 5 | *GAPDH* (5.000) | *RPS18* (3.976) | *RPS18* (4.229) | *GAPDH* (3.936) | *GAPDH* (5.000) | *RAD1a* (4.729) | *RPS18* (5.000) | *GAPDH* (5.000) | *RPS18* (5.000) |
| **Thorax** | 1 | *RPS18* (1.414) | *RAD1a* (1.189) | *RAD1a* (1.316) | *RPS5* (1.414) | *ARF1* (1.732) | *RPS18* (1.565) | *RPS18* (1.316) | *ARF1* (2.000) | *RAD1a* (1.414) |
|  | 2 | *RAD1a* (1.682) | *ARF1* (2.000) | *ARF1* (1.682) | *RAD1a* (2.213) | *RPS18* (2.000) | *ARF1* (1.682) | *RAD1a* (2.060) | *RAD1a* (2.000) | *RPS5* (1.682) |
|  | 3 | *ARF1* (2.280) | *RPS18* (2.828) | *RPS5* (2.828) | *RPS18* (2.280) | *RAD1a* (2.060) | *RPS5* (2.711) | *ARF1* (2.632) | *RPS5* (2.060) | *ARF1* (2.632) |
|  | 4 | *RPS5* (3.722) | *RPS5* (3.000) | *RPS18* (3.224) | *ARF1* (2.828) | *RPS5* (2.828) | *RAD1a* (2.828) | *RPS5* (2.828) | *RPS18* (2.449) | *RPS18* (3.224) |
|  | 5 | *GAPDH* (5.000) | *GAPDH* (5.000) | *GAPDH* (5.000) | *GAPDH* (5.000) | *GAPDH* (5.000) | *GAPDH* (5.000) | *GAPDH* (5.000) | *GAPDH* (5.000) | *GAPDH* (5.000) |
| **Gut** | 1 | *RPS5* (1.189) | *RAD1a* (1.000) | *RPS18* (1.732) | *RPS5* (1.732) | *RPS5* (1.732) | *RAD1a* (1.414) | *ARF1* (1.189) | *ARF1* (1.682) | *ARF1* (1.565) |
|  | 2 | *RPS18* (2.000) | *GAPDH* (1.682) | *GAPDH* (2.213) | *RAD1a* (2.000) | *RAD1a* (1.861) | *RPS18* (1.732) | *RAD1a* (1.861) | *RAD1a* (2.060) | *RPS18* (1.732) |
|  | 3 | *RAD1a* (2.449) | *RPS5* (3.000) | *RAD1a* (2.378) | *ARF1* (2.213) | *RPS18* (2.000) | *RPS5* (2.378) | *GAPDH* (2.449) | *RPS18* (2.590) | *RPS5* (2.213) |
|  | 4 | *ARF1* (3.464) | *RPS18* (4.000) | *RPS5* (2.783) | *RPS18* (2.632) | *ARF1* (3.130) | *ARF1* (3.464) | *RPS5* (3.722) | *GAPDH* (2.828) | *GAPDH* (3.364) |
|  | 5 | *GAPDH* (5.000) | *ARF1* (5.000) | *ARF1* (3.976) | *GAPDH* (5.000) | *GAPDH* (5.000) | *GAPDH* (5.000) | *RPS18* (5.000) | *RPS5* (3.976) | *RAD1a* (5.000) |
| **Fat body** | 1 | *RPS5* (1.189) | *RPS18* (1.189) | *RPS18* (1.189) | *RPS5* (1.189) | *ARF1* (1.414) | *RPS18* (1.565) | *RPS18* (1.414) | *ARF1* (1.565) | *RPS18* (1.316) |
|  | 2 | *RPS18* (2.000) | *RPS5* (1.414) | *GAPDH* (1.861) | *RAD1a* (2.449) | *GAPDH* (1.861) | *GAPDH* (2.213) | *RAD1a* (1.565) | *RAD1a* (1.682) | *RPS5* (2.000) |
|  | 3 | *RAD1a* (2.632) | *RAD1a* (3.224) | *ARF1* (3.344) | *ARF1* (2.632) | *RPS18* (2.913) | *ARF1* (2.913) | *RPS5* (2.449) | *RPS5* (2.711) | *GAPDH* (2.711) |
|  | 4 | *ARF1* (3.224) | *GAPDH* (3.722) | *RPS5* (3.663) | *RPS18* (2.632) | *RPS5* (3.344) | *RAD1a* (2.991) | *ARF1* (3.722) | *RPS18* (2.828) | *ARF1* (2.828) |
|  | 5 | *GAPDH* (5.000) | *ARF1* (5.000) | *RAD1a* (3.722) | *GAPDH* (5.000) | *RAD1a* (3.936) | *RPS5* (3.344) | *GAPDH* (5.000) | *GAPDH* (5.000) | *RAD1a* (5.000) |
| **Carcass** | 1 | *RAD1a* (1.565) | *RPS18* (1.316) | *RAD1a* (1.189) | *RPS5* (1.414) | *GAPDH* (1.732) | *RPS18* (1.682) | *RPS5* (1.565) | *ARF1* (1.565) | *RPS5* (1.316) |
|  | 2 | *RPS5* (2.000) | *RPS5* (2.213) | *ARF1* (1.414) | *RPS18* (1.732) | *RAD1a* (2.378) | *RAD1a* (2.213) | *RPS18* (2.000) | *RPS5* (2.000) | *RAD1a* (2.213) |
|  | 3 | *RPS18* (2.280) | *ARF1* (2.449) | *RPS18* (3.224) | *ARF1* (2.632) | *RPS5* (2.449) | *GAPDH* (2.711) | *RAD1a* (2.060) | *GAPDH* (2.280) | *ARF1* (2.449) |
|  | 4 | *ARF1* (2.828) | *GAPDH* (3.344) | *RPS5* (3.722) | *RAD1a* (3.936) | *RPS18* (2.991) | *RPS5* (2.991) | *ARF1* (3.130) | *RPS18* (3.344) | *GAPDH* (3.344) |
|  | 5 | *GAPDH* (5.000) | *RAD1a* (4.229) | *GAPDH* (5.000) | *GAPDH* (3.976) | *GAPDH* (3.344) | *ARF1* (3.344) | *GAPDH* (5.000) | *RAD1a* (4.229) | *RPS18* (4.229) |
